# Usual dietary intake estimation in the German National Cohort (NAKO)

**DOI:** 10.3389/fnut.2026.1894787

**Published:** 2026-08-26

**Authors:** Nina Wawro, Sylvia Gastell, Rafael Mikolajczyk, Nadine Glaser, Sabrina Schlesinger, Tamara Schikowski, André Karch, Henning Teismann, Nadia Obi, Volker Harth, Katharina S. Weber, Cara Övermöhle, Michael Leitzmann, Beate Fischer, Tobias Pischon, Katharina Nimptsch, Börge Schmidt, Henry Völzke, Till Ittermann, Annette Peters, Barbara Thorand, Antje Hebestreit, Maike Wolters, Verena Katzke, Stefanie Jaskulski, Peggy Sekula, Lilian Krist, Stefan N. Willich, Bernd Holleczek, Michael Hoffmeister, Carolina Klett-Tammen, Dörthe Meyerdierks, Kerstin Wirkner, Johanna Conrad, Ute Nöthlings, Matthias B. Schulze, Jakob Linseisen, Sven Knüppel

**Affiliations:** 1 Institute of Epidemiology, Helmholtz Zentrum München-German Research Center for Environmental Health (GmbH), Neuherberg, Germany; 2 Department of Epidemiology, Medical Faculty, University of Augsburg, Augsburg, Germany; 3 NAKO Study Center, German Institute of Human Nutrition Potsdam-Rehbruecke (DIfE), Nuthetal, Germany; 4 Interdisciplinary Center for Health Sciences, Institute for Medical Epidemiology, Biometrics, and Informatics, Medical Faculty of the Martin-Luther University Halle-Wittenberg, Halle, Germany; 5 German Diabetes Center, Leibniz Center for Diabetes Research at Heinrich Heine University, Institute for Biometrics and Epidemiology, Düsseldorf, Germany; 6 German Center for Diabetes Research (DZD), Neuherberg, Germany; 7 IUF-Leibniz Research Institute for Environmental Medicine, Düsseldorf, Germany; 8 Institute of Epidemiology and Social Medicine, University of Münster, Münster, Germany; 9 Institute for Occupational and Maritime Medicine (ZfAM), University Medical Center Hamburg-Eppendorf, Hamburg, Germany; 10 Institute of Epidemiology, Kiel University, Kiel, Germany; 11 Department of Epidemiology and Preventive Medicine, University of Regensburg, Regensburg, Germany; 12 Molecular Epidemiology Research Group, Max Delbrück Center for Molecular Medicine in the Helmholtz Association (MDC), Berlin, Germany; 13 Biobank, Max Delbrück Center for Molecular Medicine in the Helmholtz Association (MDC), Berlin, Germany; 14 Charité - Universitätsmedizin Berlin, Corporate Member of Freie Universität Berlin and Humboldt-Universität zu Berlin, Berlin, Germany; 15 Institute for Medical Informatics, Biometry, and Epidemiology, University Hospital Essen, Essen, Germany; 16 Institute for Community Medicine, University Medicine Greifswald, Greifswald, Germany; 17 Institute for Medical Information Processing, Biometry, and Epidemiology, Ludwig-Maximilians-Universität München, Munich, Germany; 18 Leibniz Institute for Prevention Research and Epidemiology - BIPS, Bremen, Germany; 19 Division of Cancer Epidemiology, German Cancer Research Center, Heidelberg, Germany; 20 Institute of Epidemiology and Prevention, Faculty of Medicine and Medical Center – University of Freiburg, Freiburg, Germany; 21 Institute of Social Medicine, Epidemiology and Health Economics, Charité-Universitätsmedizin Berlin, Berlin, Germany; 22 Saarland Cancer Registry, Saarbrücken, Germany; 23 Division of Clinical Epidemiology and Aging Research, German Cancer Research Center (DKFZ), Heidelberg, Germany; 24 Department of Epidemiology, HZI-Helmholtz-Zentrum für Infektionsforschung GmbH, Braunschweig, Germany; 25 Leipzig Research Center for Civilization Diseases (LIFE), Leipzig University, Leipzig, Germany; 26 German Nutrition Society, Bonn, Germany; 27 Institute of Nutritional and Food Sciences, University of Bonn, Bonn, Germany; 28 Department of Molecular Epidemiology, German Institute of Human Nutrition Potsdam-Rehbruecke (DIfE), Nuthetal, Germany; 29 Institute of Nutritional Science, University of Potsdam, Nuthetal, Germany; 30 Department Food and Feed Safety in the Food Chain, Unit Human Study Center Consumer Health Protection, German Federal Institute for Risk Assessment (BfR), Berlin, Germany

**Keywords:** 24-h food list, food frequency questionnaire, measurement error correction, multiple source method, nutritional epidemiology, population-based cohort

## Abstract

**Introduction:**

Accurate measurement of dietary intake remains challenging in large-scale nutritional studies. This study aimed to develop and evaluate both a practical dietary assessment strategy and a computationally efficient statistical method for estimating usual dietary intake in the German National Cohort (NAKO Gesundheitsstudie).

**Methods:**

We developed a blended approach using data from NAKO. During baseline (2014–2019) and first follow-up examinations (2019–2024), up to four 24-h food lists (24 h-FLs) and one food frequency questionnaire (FFQ) were collected. We combined these dietary intake data sources using an adapted Multiple Source Method (MSM) and supplemented them with estimated consumption amounts based on data from the German National Nutrition Survey II (NVS II, 2005–2007) to generate measurement-error-corrected estimates of dietary intake. The adapted MSM was empirically evaluated against conventional logistic linear mixed-effects model (LLMM), which can be computationally complex for large datasets due to lengthy processing times. Additionally, a simulation study evaluated how varying the number of 24 h-FLs and FFQ assessments affected the consumption probability estimates.

**Results:**

The adapted MSM showed high statistical agreement with LLMM (correlation ≥0.97). The usual intake of 90 EPIC-SOFT food groups, 124 nutrients, and energy intake was estimated for 152,304 participants (75% of the cohort) who had at least one 24 h-FL and an FFQ available. Furthermore, the simulation showed that including repeated 24 h-FLs alongside an FFQ improved the accuracy of individual consumption probability estimates, particularly when only one or two 24 h-FLs were available.

**Conclusion:**

The adapted MSM offers a computationally efficient, practical alternative to LLMM, generated dietary intake estimates for over 150,000 NAKO participants to support future research. By integrating repeated 24 h-FLs, an FFQ, and external consumption data, this blended approach balances logistical feasibility with statistical precision, providing a scalable, cost-effective framework for large-scale nutritional studies.

## Introduction

A growing body of evidence shows that diet plays a crucial role in preventing and managing chronic non-communicable diseases (NCDs), such as cardiovascular diseases, cancer and diabetes (1–3). This underscores the need to understand how dietary patterns link to diseases and other health outcomes, necessitating accurate dietary pattern assessments. However, collecting accurate data on dietary intake in large-scale epidemiological studies remains difficult.

Common dietary assessment instruments such as food records, 24-h dietary recalls and food frequency questionnaires (FFQs) have inherent limitations, including participant burden and recall bias (4, 5). Prospective food records require a high degree of participant motivation and can alter habitual eating behaviors due to reactivity. While traditional interviewer-administered 24-h dietary recalls provide rich detail, they demand trained staff, which has historically made them economically prohibitive for large-scale epidemiological cohorts. Automated, self-administered digital 24-h recalls (e.g., ASA24) have largely mitigated this interviewer burden. However, FFQs remain widely used despite being prone to substantial recall bias and challenges in cognitive averaging. These inaccuracies can lead to measurement errors that weaken the findings of studies on the relationship between diet and health (6, 7). Therefore, developing participant-friendly approaches for dietary assessment and establishing valid statistical methods for the estimation of usual dietary intake are crucial needs in nutritional epidemiology.

The German National Cohort (NAKO, “NAKO Gesundheitsstudie”) is an ongoing large-scale multi-center, prospective, population-based cohort study. It is characterized by a highly comprehensive nature of its examination program, including dietary assessment (8). Consequently, a computationally efficient statistical approach was required to handle the large volume of dietary data collected within the cohort.

Based on the assumption that the probability of consumption for certain foods has a greater influence on variation in usual dietary intake than do differences in portion size (9), a blended dietary assessment approach was developed. The design combined two complementary dietary assessment instruments: repeated 24-h food lists (24 h-FLs) and an FFQ to estimate dietary consumption probabilities (10–12). Specifically, the 24 h-FL assessed dietary intake from the previous day but omitted detailed quantities and portion sizes, thereby substantially reducing participant burden compared to a traditional 24-h dietary recall. Traditional 24-h dietary recalls provide continuous quantitative data, whereas the 24 h-FL provides binary (yes/no) consumption data and thus generates frequencies rather than absolute consumption quantities. We adopted the Multiple Source Method (MSM) to statistically model these repeated binary data for this specific study design (13). These frequencies can be used in combination with an external source, such as the German National Nutrition Survey II (NVS II) (14), which is useful for estimating intake amounts or portion sizes, to thereby estimate usual dietary intake (15).

The primary aim of this study was to provide a detailed description and systematic evaluation of the innovative approach utilized for estimating the usual dietary intake of NAKO participants, offering a tool applicable to other large-scale nutritional studies. Specifically, our research aims were twofold: (1) to present the algorithm developed to estimate usual dietary intake by combining data from two dietary assessment instruments (the 24 h-FL and FFQ) along with data from the NVS II (15), and (2) to provide a computationally efficient statistical framework for large-scale nutritional studies.

## Materials and methods

### Study population

By combining data from the baseline (2014–2019) and first follow-up (2019–2024) of NAKO study, a total of 204,706 participants (103,284 women and 101,422 men) aged 19 to 74 years were included. Participants provided data at either one or both time points. To handle analytical overlap and maximize the use of available data, observations from the baseline and follow-up were pooled cross-sectionally, treating the questionnaires as clustered repeated measures within each participant.

Participants were drawn from age- and sex-stratified random samples from compulsory registries of residents within the 16 defined study areas across the 18 German study centers. A detailed description of the extensive examination program can be found elsewhere (8, 16). Written informed consent was obtained from all participants.

The NAKO study is performed with the approval of the relevant ethics committees and in accordance with national laws and with the Declaration of Helsinki of 1975 (in its current, revised version). The NAKO study is registered in the German Clinical Trials Register (DRKS) under the identifier DRKS00037328.

### Dietary assessment

The nutrition module of the NAKO comprised up to four 24 h-FL^1^ and one FFQ^2^ to be completed at home, except for the first 24 h-FL that was completed during the study center visit. All questionnaires were available online, but paper versions were handed out upon request. Both questionnaires were provided by the German Institute of Human Nutrition Potsdam-Rehbruecke (DIfE). Additionally, adherence to vegan or vegetarian dietary habits was assessed in the FFQ. This information was used later in the analytical pipeline to perform plausibility checks and zero-imputation when evaluating the consumption probabilities of animal-derived products.

The 24 h-FL was designed to self-report the consumption of pre-specified foods during the last 24 h (11). The 24 h-FL comprised 27 main food groups. Whenever the main question of consumption was affirmed (e.g., did you eat bread or bread roll yesterday?), the participant was asked to specify from a more detailed list what she or he had consumed (e.g., whole-grain bread, multi-grain bread, wheat bread, etc.). The 24 h-FL only assessed if a certain item was consumed (yes or no) but the amount of consumption was not specified. The estimated time for completion was 10 min (11). The participants were asked to fill in the first 24 h-FL during their study center visit. The second to fourth 24 h-FLs were planned to be completed every 3 months at home following an invitation from the study center.

Although the 24 h-FL had not been formally validated, it was considered appropriate for the NAKO study, as its food list had been developed using data from the nationally representative NVS II survey. It was chosen as the primary instrument because it enabled repeated dietary assessments in large cohorts while minimizing the respondent burden commonly associated with other short-term methods, such as 24-h dietary recalls and food records. In addition, it was expected that the direct yes/no format for the consumption questions would be easily understandable and pose few cognitive barriers to the participants. In a feasibility study, more than 90% of participants found the 24 h-FL to be good or very good in terms of its understandability, usability and visual presentation. The optimal number of repeated 24 h-FLs required to generate mathematically stable consumption probability estimates was evaluated in a simulation study, which is included in this article.

To provide a long-term measurement, the FFQ (12) was used to assess the intake of foods during the past 12 months. It asked for the frequency of consumption of a closed list of food items, presented with a specified portion size. Additionally, the intake of dietary supplements, the avoidance of certain foods or recent changes in diet were assessed. Within a main food group (e.g., fruits and vegetables) the frequency of consumption of single items (e.g., a banana, a handful of grapes) or combined items (e.g., an apple or a pear) had to be categorized, ranging from “never” and “once a month or less” to “daily” or even more frequent. The frequency categories provided for selection differed per food item. The participants were asked to complete the FFQ after their study center visit, and if possible, on the same day.

In the German part of the EPIC study, a version of the FFQ prior to the one that was used in the NAKO study had been compared to 24-h dietary recalls and showed correlations of 0.49 to 0.89 (median = 0.70) when comparing the FFQ food groups with food intake assessed by 24-h dietary recalls (17). These correlations were somewhat higher than those reported in a systematic review on FFQ validity, which found correlations ranging from 0.22 to 0.77 (median = 0.42) for energy intake and nutrients (18).

Although the target was set at two to four 24 h-FLs and one FFQ per participant, only around 61% of participants had completed at least one 24 h-FL and the FFQ following the baseline assessment. To address this shortfall and achieve the target of four 24 h-FLs and one FFQ, participants were invited to complete the nutrition assessments during their first follow-up visit at the study centers, approximately 5 years after the baseline examination. All completed 24 h-FLs and the corresponding FFQ were analyzed collectively, without distinction between the baseline and follow-up assessment in which they were completed. Participants were included in the final analysis if they had provided at least one 24 h-FL and the FFQ each of which was at least 70% complete. This criterion applied regardless of whether the assessments were completed during the baseline or first follow-up.

### Assessment of covariates

A uniform set of adjustment variables was selected for all statistical models. This ensured consistent adjustment when estimating both food item consumption probabilities in the NAKO data and person-specific consumption-day amounts in the NVS II data. Analyzing the NVS II data showed that the five most frequently selected variables for estimating the parameters for person-specific consumption-day amounts were age, sex, smoking status, years of education and body mass index (BMI) (19). These five variables from the baseline assessment were used as a uniform set of adjustment variables. We assumed that any changes in the covariates by the time of the follow-up occurred at random and did not affect the estimation of the usual dietary intakes. Age (years) was computed as the date of examination minus the date of birth. Sex (male/female) was reported in the main questionnaire. Education was grouped into three categories, representing low, moderate and high education levels based on the highest self-reported education level (ISCED 2011): no degree, in training or vocational education (level 0 to 4), professional school or similar degree (level 5 & 6), and university degree or higher (level 7 & 8). BMI (kg/m^2^) was calculated from measured body weight and height. Self-reported smoking status was categorized as current, never and former smoker. Thus, age and BMI were modeled as continuous variables, while sex (women and men), education (low, moderate, and high), and smoking status (current, never, and former) were modeled as categorical variables. These covariates were utilized in both stages of the dietary assessment framework: first, to adjust the consumption probability in the logistic part of the MSM model using NAKO data, and second, to adjust the consumption amounts within the linear mixed-effects models using the external NVS II data. In the statistical modeling part of the consumption probabilities, we also adjusted for food frequency from the FFQ and the study center to which the participant was assigned.

The proportion of missing values in covariates ranged from 0.5% (BMI) to 4% (education level). Given the low proportion of missing data, we used single imputation with predictive mean matching. While multiple imputation is generally recommended because it accounts for imputation uncertainty, the impact of using single imputation is expected to be negligible in this setting. In addition, single imputation was selected as a pragmatic approach because it yields a single set of estimated usual dietary intakes that can be readily incorporated into subsequent analyses and shared for future research applications.

### Statistical modeling of usual dietary intake

For the purposes of modeling of usual dietary intake, “consumption probability” was defined as the statistical probability (ranging from 0 to 1) that a specific individual consumes a single food item on any given randomized day. “Consumption-day amounts” are defined as the absolute quantitative volume (in grams) of that food item consumed by the individual, conditional on it being a day where the item is consumed. The usual dietary intake (g/day) of a single food item was derived by multiplying the estimated consumption probability and the estimated person-specific consumption-day amount of that food item.


$$\begin{array}{l} \mathrm{Usual}\;\mathrm{intake}\;\mathrm{of}a\mathrm{food}\;\mathrm{item}=\mathrm{consumption}\;\mathrm{probability}\\ \;\;\;\;\;\;\;\;\;\;\;\;*\mathrm{consumption}-\mathrm{day}\mathrm{amount} \end{array}$$

We calculated the usual food group and nutrient intakes using the model-then-add approach (20), which involved separately modeling the usual intake of each food item and then summing up these estimates to determine the usual intake for the respective food groups or nutrients.

**Figure 1:**
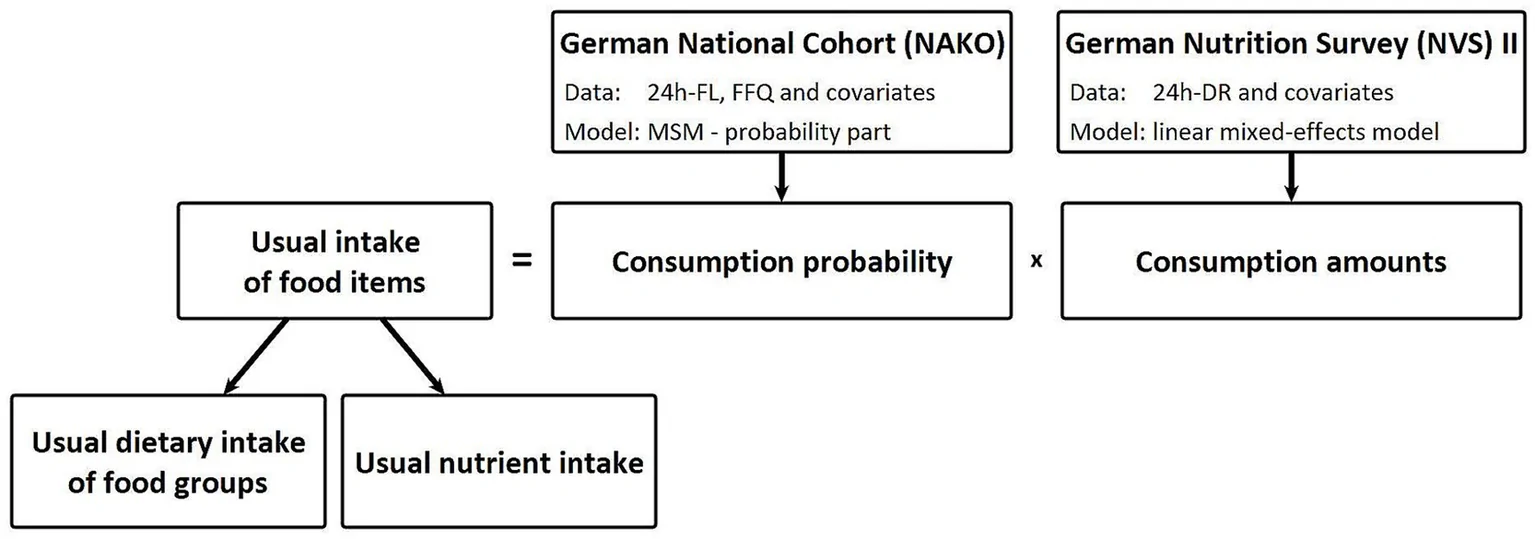
Overview of design to estimate usual dietary intake in NAKO.

The following paragraphs describe in detail the steps we carried out to derive the consumption probabilities and the person-specific consumption-day amounts as parts of estimating usual dietary intake, as depicted in Figure 1.

### Estimation of dietary consumption probabilities

As there was no established gold standard for estimating dietary consumption probabilities for a certain food item, the estimation was based on 24 h-FLs, as it was assumed that 24 h-FLs are an unbiased source of information for this estimation.

To model the dietary consumption probability of a food item for a participant, we adapted the probability part of the MSM method (13). For the probability component, an indicator variable $p_{ij}$ was assigned a value one if the participant i consumed the considered food item on day j in a 24 h-FL; otherwise, it was assigned a value of zero $\left(p_{ij}\in \left\{0,1\right\}\right)$ . The total number of participants was denoted n . The total number of 24 h-FL was denoted $M=\sum_{i=1}^{n}n_{i}$ , where $n_{i}$ was the number of 24 h-FLs of individual i . The predicted dietary consumption probability was denoted by $m_{ij|Z}$ for participant i . Using a logistic regression model with a logit-link the adjusted predicted consumption probability $m_{ij|Z}$ was modeled by incorporating a set of adjustment variables Z (such as age, sex and frequency information from an FFQ) but did not include variables of the sampling day (e.g., season or weekday/weekend day). This resulted in the partition:


$$p_{ij}=m_{i|Z}+r_{ij}$$

where $m_{i|Z}=\mathrm{Prob}\left(p_{ij}=1|z,\beta \right)={\left\{1+\exp\left(\sum_{r=1}^{k+1}\beta_{r}z_{ir}\right)\right\}}^{-1}\in \left(0,1\right)$ was the predicted consumption probability for individual i with $z_{i1}=1$ as a constant and $z_{ir}$ $\left(i=1\ldots ,n;r=2,\ldots ,k+1\right)$ on k adjustment variables and $\beta_{r}\left(r=1,\ldots ,k+1\right)$ referred to maximum likelihood estimates from the logistic regression model.

The corresponding residuals were $r_{ij}=p_{ij}-m_{i|Z}\in \left(-1,1\right)$ . In contrast to the original version of the MSM, the residuals were not transformed, so that the need for an back transformation after the shrinkage was no longer necessary.

The residuals $r_{ij}$ were further decomposed into two components:


$$r_{ij}=t_{i}+u_{ij}$$

where $t_{i}$ represented the expected value for individual *i* with an expectation *E* ( $t_{i}$ ), while $u_{ij}$ captures the day-to-day variation for individual *i* with mean zero. The two components were assumed to be independent, with variances $\sigma_{t}^{2}$ and $\sigma_{u}^{2}$ , respectively.

Applying shrinkage on the residuals resulted in


$$r_{i}^{*}=\kappa \left({\bar{r}}_{i\cdot }-{\bar{r}}_{\cdot \cdot }\right)+{\bar{r}}_{\cdot \cdot }$$

where $\kappa =\left(\sigma_{t}^{2}/\left(\sigma_{t}^{2}+\left(\sigma_{u}^{2}/n_{i}\right)\right)\right)$ was the shrinkage factor, $r_{i}^{*}$ the mean of the residuals of individual i and ${\bar{r}}_{\cdot \cdot }=\left(1/M\right)\sum_{i=1}^{n}\sum_{j=1}^{n_{i}}r_{ij}$ the population mean. The shrinkage factor κ mitigated (day-to-day) intra-individual variation by shifting an individual’s estimate toward the population mean, providing a more reliable estimate than naïve averaging the repeated measurements. The variance components $\sigma_{t}^{2}$ and $\sigma_{u}^{2}$ were estimated by using the formulas given for one-way analysis of variance (random intercept model) (21), which are shown in Supplementary material. Finally, the probability of consumption $p_{i}^{*}$ for participant i was estimated by


$$p_{i}^{*}=m_{ij|Z}+r_{i}^{*}$$

In case of convergence problems of the logistic regression model, we applied Firth’s logistic regression with an added covariate (FLAC) (22).

Particularly noteworthy is that the estimated probability of consumption $p_{i}^{*}$ did not assume that all participants had the same number of repeated 24-h FLs. The algorithm took account of the varying number of observations per participant (which ranged from 1 to 4 per participant) by incorporating the specific number $n_{i}$ both into the estimation of the variance components and into the calculation of the shrinkage factor at the individual level. Supplementary material contains R and SAS code to apply the MSM probability part as described here.

### Estimation of dietary consumption-day amounts

Because the 24 h-FLs did not gather consumption-day amounts for food items, these amounts had to be estimated from an external study population. Within the NVS II, linear mixed effects models were applied to estimate individual consumption-day amounts based on two repeated 24-h dietary recalls (14). This allowed the transfer of the estimated model parameters to the NAKO participants to predict individual daily person-specific consumption-day amounts for each food item. The 24-h dietary recalls of the NVS II were assumed to enable unbiased estimates of the person-specific consumption-day amounts.

### Estimation of usual dietary intake

The usual intake of a single food item was derived by multiplying the consumption probability and the person-specific consumption amount of this item. The single food items were grouped according to the EPIC-SOFT food groups (23), resulting in 73 food subgroups and 17 main food groups (see Supplementary Table S1).

The 124 micro- and macro-nutrients were obtained by linking the estimated individual usual intake amounts of the single food items to the German food composition database (Bundeslebensmittelschlüssel, version 3.02) and summing up nutrients across all food items for each participant.

In a final step, participants who reported no intake of a certain food item in all of the up to four 24 h-FLs and reported to “never” consume that item in the FFQ were identified as non-consumers. Hence, the estimated usual intake of that food item was set to zero.

As a plausibility check, we compared the usual intake estimates to results from the Kooperative Gesundheitsforschung In der Region Augsburg (KORA) FF4 study that applied a comparable approach. An adjusted LLMM was used to estimate consumption probabilities in this Bavarian study, using repeated 24 h-FLs and an FFQ (24).

### Empirical evaluation: comparison of adapted MSM method and logistic linear mixed-effects model (LLMM) to estimate consumption probabilities

We evaluated the adapted MSM method for predicting individuals’ consumption probability of a single food item from repeated 24 h-FLs by comparing its predictions to those from a logistic linear mixed-effects model using the SAS PROC GLIMMIX (25). The LLMM was a conventional method to model binary repeated measurements. The NAKO sample size led to computational challenges prohibiting the application of the LLMM. To assess the validity of our estimates, we compared results from the adapted MSM and the LLMM in a random subsample of 5,000 NAKO participants, since applying the LLMM to the full sample was infeasible. Generally, applying the LLMM on data sets with a high number of participants (clusters, random effects), several repeated measures on each participant (within-cluster units) and several adjustment variables leads to substantial computational challenges.

We compared three food items—buttermilk, tomatoes and grapes—chosen for their varying average consumption frequencies ranging from frequent (tomatoes, 50%) over intermediate (grapes, 25%) to rare (buttermilk, 2%). These specific foods were selected to reflect different consumption patterns, ranging from frequently consumed foods to those consumed only occasionally, in order to thoroughly test the performance of the MSM and the boundary conditions under various statistical parameters. The LLMM incorporated a random intercept and was fitted using adaptive Gauss-Hermite quadrature. Example code for the application in R and SAS is given in Supplementary material. The methodology of the LLMM is also used in the consumption probability estimation of the NCI method, which is one of the few methods available for estimating the usual dietary intakes (26).

### Simulation study: the effect of the number of 24 h-FL and the food frequency from FFQ on estimating consumption probabilities

The simulation study was designed to assess the influence of the number of observed 24 h-FLs and the food frequency from the FFQ on estimating the consumption probability. We investigated the same three food items (namely buttermilk, tomatoes and grapes) as in the empirical evaluation. For a source population of size *N*_source_ = 200,000, we simulated the true frequency (yes or no on each simulated day) of a certain food item in 365 days. For each day, a binary variable was drawn from a binomial distribution, with the true individual p chosen as the consumption probability estimate derived within the NAKO. As the 24 h-FL did not record amounts consumed, we focused the simulation study on the estimation of the consumption probability, not modeling the amounts. The true FFQ information was derived from the simulated true observations by evaluating consumption frequencies for every individual. That is, when consumption was simulated for an individual on *x* out 365 days, the true FFQ would be *x*/365. The simulated consumption frequencies were grouped according to the FFQ applied in the NAKO. For example, for *x* = 12 days, this would result in 12 times per year (12/365), or “once a month.” As there is often misreporting in FFQs, we added an error to the true FFQ value by assuming the probability of misreporting a wrong category was up to 50%, where misreporting to an adjacent category was more likely than more extreme misreporting.

From this simulated population, we sampled *N*_sample_ = 20,000 individuals without replacement as random draws. This sample size was chosen to ensure a fairly high number of individuals and at the same time not to make the computing time for each simulation run too long. For every simulated individual, *n*_recalls_ = 2, 4, 5, 6, 8, 10 and 12 of 24 h-FL observations were randomly drawn out of the available 365 observations. Based on these sampled 24 h-FLs, the consumption probability per individual $\hat{p_{i}}$ was estimated using the MSM method as described above. Models were fitted with and without including the food frequency information from the simulated FFQ in addition to the covariates as they were observed within the NAKO. All models were fitted *n*_rep_ = 100 times. In addition, squared partial correlation of the FFQ frequency and the true consumption probability was examined, accounting for the covariates.

We compared the influence of the number of observed 24 h-FLs for three different food items, characterized by their mean consumption probability. Tomatoes were most frequently consumed (mean consumption probability of 0.5), whereas grapes (0.25) had a moderate consumption probability and buttermilk with a mean consumption probability of 2% was only rarely consumed.

To assess the deviations between individuals’ probability estimates $\hat{p_{i}}$ , i=1,…,20000 , and the simulated true consumption probability $p_{i}$ , we evaluated the mean absolute deviations to the true individual consumption probability $\mathrm{MAD}=\left(1/N\right)\sum_{i=1}^{N}\left|\hat{p_{i}}-p_{i}\right|$ and the squared correlation $\mathrm{Corr}=\left(\hat{p_{i}},p_{i}\right)$ for each simulation run. Both measurements were averaged over the 100 replications.

All calculations were carried out using R (version 4.2.2) or SAS (version 9.4), utilizing SAS Enterprise Guide (version 8.1).

## Results

From the 152,304 participants who provided sufficient dietary information, 78,252 (51.4%) were women and the mean age was 50.0 years (standard deviation (SD) = 12.6). The mean body mass index measured was 26.5 kg/m^2^ (SD = 5.0). Demographic characteristics of the analyzed sample and the total NAKO cohort are presented in Table 1.

**Table 1:** Population and sample characteristics.

Characteristic	Total sample (*n* = 204,706)		Sample with usual intake estimates available (*n* = 152,304)	
	Mean	SD	Mean	SD
Age (years)	49.9	12.8	50.0	12.6
BMI (kg/m^2^)	26.7	5.1	26.5	5.0
	*n*	%	*n*	%
Sex				
Female	103,284	50.5	78,252	51.4
Male	101,422	49.5	74,052	48.6
Education				
High	47,405	23.2	38,221	25.1
Medium	64,658	31.6	49,619	32.6
Low	92,643	45.3	64,464	42.3
Smoking				
Never smoker	94,238	46.0	72,911	47.9
Current smoker	41,976	20.5	27,643	18.2
Former smoker	68,492	33.5	51,750	34.0

SD, standard deviation; BMI, body mass index.

In total, 458,049 24 h-FLs from the baseline assessment and the first follow-up from 204,706 participants were available. Thereof, 444,839 (97.1%) were completely filled, 454,659 (99.3%) were filled in to at least 90% and 457,128 (99.8%) were filled in to at least 70%. Regarding the FFQ, out of 154,855 participants who started to fill in the questionnaire, 138,300 (89.3%) filled it in completely, 152,417 (98.4%) completed at least 90% and 153,489 (99.1%) participants completed at least 70%. Finally, we used data from 152,304 participants comprising 438,166 24 h-FLs. The mean number of filled in 24 h-FLs per participant included in the analysis was 2.9 (range 1 to 4).

Table 2 shows in detail the numbers of usable questionnaires across the 18 study centers. 25.0% of all participants did not provide sufficient information in the FFQ and 17.6% of all participants did not provide any sufficiently completed 24 h-FL. A variation in response between the study centers was observed. Overall, of the 153,489 participants with a useable FFQ, 27,505 (18.1%) had only one useable 24 h-FL, 35,305 (23.2%) had only two useable 24 h-FLs, 17,925 (11.8%) had only three useable 24 h-FL and 71.569 (47.0%) had four useable 24 h-FL available.

**Table 2:** Number of participants by study center: number of usable (more than ≧70% filled in) 24-h food lists (24 h-FLs) and food frequency questionnaires (FFQ).

	FFQ usable (more than ≧70% filled in)												
	Yes						No						
	Number of 24 h-FL per participant (more than ≧70% filled in)					Sum	Number of 24 h-FL per participant (≥70% filled in)					Sum	Total
Study center	0	1	2	3	4	*N*	0	1	2	3	4	*N*	*N*
Augsburg	49	1,597	4,897	2,910	8,011	**17,464**	734	1,599	632	61	107	**3,133**	**20,597**
Regensburg	23	1,038	1,910	963	4,756	**8,690**	868	336	94	14	12	**1,324**	**10,014**
Heidelberg-Mannheim	34	1,841	1,615	758	3,889	**8,137**	1,649	392	64	10	16	**2,131**	**10,268**
Freiburg	13	590	1,222	493	4,035	**6,353**	500	2,987	177	22	18	**3,704**	**10,057**
Saarbrücken	24	1,167	1,269	710	3,149	**6,319**	3,431	240	19	1	—	**3,691**	**10,010**
Essen	9	1,427	2,202	1,036	2,781	**7,455**	1,079	1,871	190	21	15	**3,176**	**10,631**
Münster	16	1,334	1,460	746	2,504	**6,060**	3,589	312	37	1	—	**3,939**	**9,999**
Düsseldorf	32	1,344	1,566	665	2,567	**6,174**	2,652	218	40	2	2	**2,914**	**9,088**
Halle	13	1,918	2,120	1,775	1,950	**7,776**	1,437	729	153	20	5	**2,344**	**10,120**
Leipzig	136	1,333	1,150	620	6,101	**9,340**	1,145	282	53	7	23	**1,510**	**10,850**
Berlin-Nord	9	1,298	1,761	995	4,302	**8,365**	1,447	215	26	3	2	**1,693**	**10,058**
Berlin-Mitte	38	1,741	2,138	801	3,925	**8,643**	2,008	303	43	4	7	**2,365**	**11,008**
Berlin-Süd	1	538	682	476	7,077	**8,774**	335	663	146	24	35	**1,203**	**9,977**
Braunschweig-Hannover	102	1,422	1,721	649	2,312	**6,206**	3,550	216	30	9	4	**3,809**	**10,015**
Hamburg	466	3,069	2,988	1,182	874	**8,579**	1,077	366	36	3	—	**1,482**	**10,061**
Bremen	24	886	1,614	855	4,355	**7,734**	2,293	368	69	13	9	**2,752**	**10,486**
Kiel	17	1,282	1,748	744	3,708	**7,499**	1,648	296	34	3	6	**1,987**	**9,486**
Neubrandenburg	179	3,680	3,242	1,547	5,273	**13,921**	6,544	1,096	265	61	94	**8,060**	**21,981**
Total, *N*	**1,185**	**27,505**	**35,305**	**17,925**	**71,569**	**153,489**	**35,986**	**12,489**	**2,108**	**279**	**355**	**51,217**	**204,706**
Total, %	**0.58**	**13.44**	**17.25**	**8.76**	**34.96**	**74.98**	**17.58**	**6.1**	**1.03**	**0.14**	**0.17**	**25.02**	**100**

Values in bold represent the total number of participants and column percentages.

Means and standard deviations of usual intakes of the main food groups, energy and macro-nutrients are presented in Table 3 stratified by sex and compared to results from the KORA FF4 study. Overall, a good accordance between studies was observed. Among the 78,252 women, the mean usual intake of dairy products, potatoes, meat and meat products, fats, sugar and sweets, cake and alcoholic drinks was considerably lower than in the 74,052 men. In contrast, a higher mean usual intake was observed for vegetables and fruits and nuts. A mean total energy intake of 1,488 kcal/day was observed in women and 2,053 kcal/day in men. Mean macro-nutrient intakes reflect the usual dietary intakes of food groups: women consumed less alcohol (4.2 g/day compared to 10.4 g/day), less protein (52.3 g/day compared to 73.7 g/day) and less carbohydrates (164 g/day compared to 215.8 g/day) compared to men.

**Table 3:** Usual dietary intake of the main food groups, energy and macronutrients per day in NAKO participants compared to KORA FF4 participants by sex.

	Female			Male		
	NAKO		KORA-FF4	NAKO		KORA-FF4
Food and nutrient intake	78,252		830	74,052		772
	Mean	SD*	Mean	Mean	SD*	Mean
Main EPIC-SOFT food groups (g/day)						
Potatoes	44.8	15	55.4	59.2	18.7	66.3
Vegetables	166.0	54.3	192.3	148.5	50.8	156.7
Legumes	6.8	3.6	6.8	6.9	3.5	5.4
Fruits and nuts	273.0	110.7	176.1	241.5	105.9	167.2
Dairy products	187.4	82.3	216.4	202.5	104.4	174.5
Cereals	115.0	30.3	144.1	179	41	193.7
Meat and meat products	49.9	22.7	89.2	107.1	41.1	144.5
Fish	15.5	9.8	18.4	21.6	13.9	23.6
Egg and egg products	17.5	9.7	16.0	22.9	12.7	18.6
Fats	11.4	3.6	20.9	19.8	6.3	29.3
Sugar and sweets	28.6	12.9	35.4	37.7	17.7	39.7
Cake	35.5	14.1	49.4	43.6	18.8	58.5
Non-alcoholic drinks	2011.2	354.5	1588.7	1927.5	369.8	1513.8
Alcoholic drinks	54.9	52.3	70.9	198.2	163.1	336.9
Energy and macronutrients intake per day						
Total energy intake (kcal)	1487.6	287	1620.2	2053	364.4	2122.3
Alcohol (g)	4.2	4.1	4.5	10.4	8.4	15.8
Dietary fiber (g)	20.0	5.6	17.4	22.6	6.1	18.4
Protein (g)	52.3	10.1	62.1	73.7	13.5	78.2
Fats (g)	63.7	13.6	68.5	89.1	17.0	88.3
Carbohydrate (g)	164	35.3	177.4	215.8	44.5	222.6

*SD, standard deviation.

The usual dietary intake estimates were derived from differing numbers of 24 h-FL available for each individual. For 27,505 (18.1%) individuals, only one 24 h-FL was available next to the FFQ. During deriving usual dietary intake estimates and quality checking, we examined macro nutrient estimates in subgroups having at least two, at least three and four 24 h-FL available. We found that for total energy, alcohol, fiber, protein, fat and carbohydrates estimated mean intakes were lower whenever participants with less available 24 h-FLs were included (see Supplementary Table S2).

Applying MSM and LLMM with the three selected food items in the simulated subsample resulted in correlations (*R*^2^) of 0.97, 0.98 and 0.99 for buttermilk, grapes and tomatoes, respectively. Scatter and density plots can be found in Supplementary Figure S1, which underpin the high correlation between the two statistical approaches.

**Figure 2:**
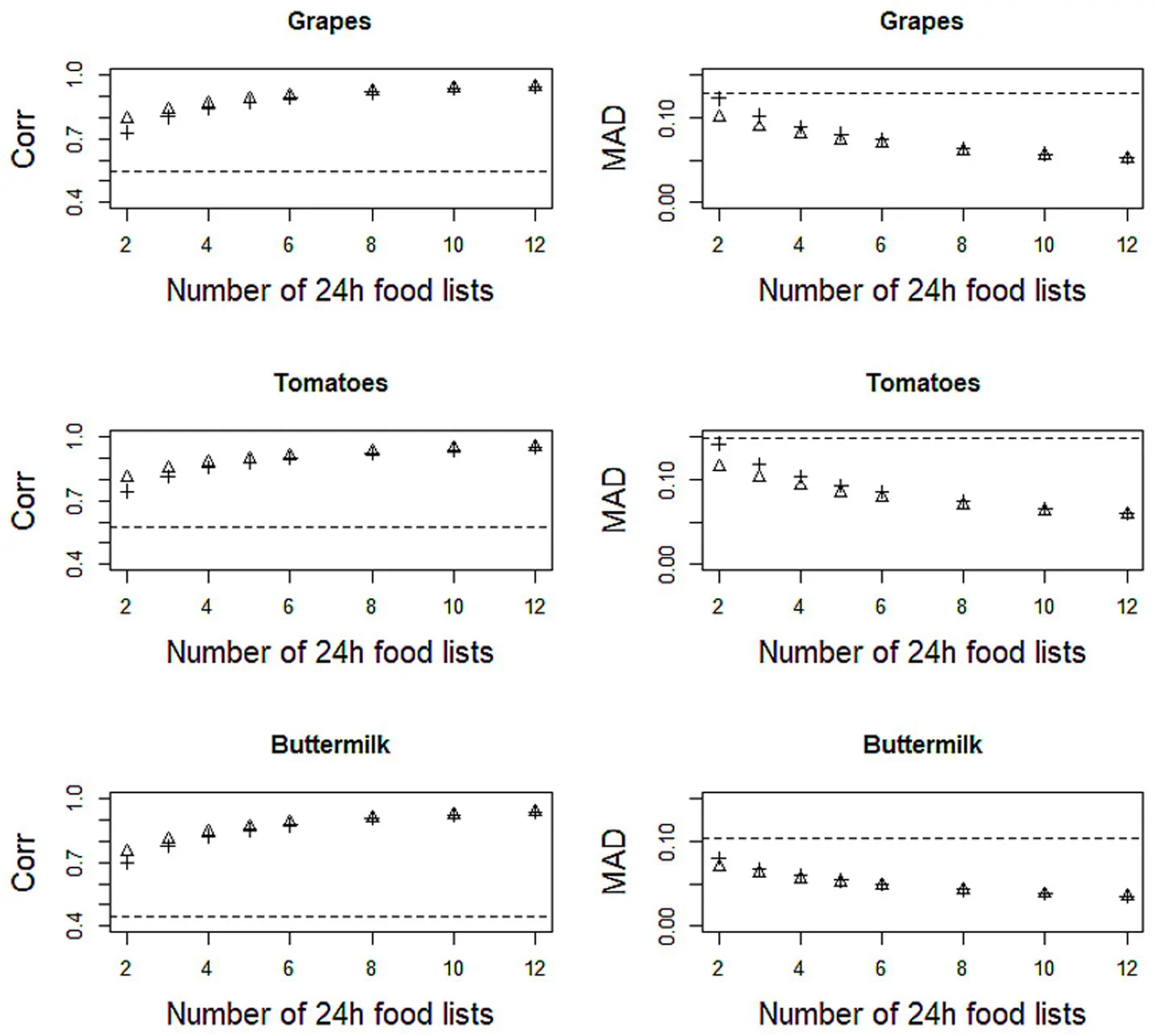
Comparison of squared correlations (Corr) and mean absolute deviations (MAD) for different food items when varying information is included in estimating consumption probabilities. Cross: model with covariates only. Triangle: model with covariates and FFQ, Dashed line: model with FFQ only. The included covariates were age at examination, sex, education, smoking status and body mass index.

Table 4 and Figure 2 shows the results of the simulation study, comparing the different food items and the information included when estimating the consumption probability model. For all three different food items, the impact of the FFQ vanished with an increasing number of available 24 h-FLs. The models with and without adjustment for FFQ frequency were most different when two replicates of 24 h-FLs were used. As the number of replicates increases toward twelve, the models converge, indicating that while the FFQ provides a vital accuracy boost when short-term data is limited, its added benefit diminishes as more 24 h-FLs for each participant were available. When the number of available 24 h-FL is limited, the benefit of the food frequency information was apparent as the correlation reported increased by at least 5%: for example, for tomatoes, the correlation increased from 0.74 to 0.81 when adding the FFQ information to two available 24 h-FL. Relying on the FFQ information only was not superior to relying on at least two 24 h-FL plus covariate information (food frequency information, age, sex, body mass index, education, smoking status and study center). For tomatoes, grapes and buttermilk, squared partial correlation coefficients were 0.58, 0.55 and 0.45 (FFQ information only), in contrast to squared correlations coefficients of 0.74, 0.73 and 0.70, respectively, for the model without FFQ information and two 24 h-FLs completed. Correlations were always higher in the models including covariates than compared to the partial correlation of the FFQ information only. The results were similar for all three selected food items, suggesting that no gradient of increasing information was observed here for foods with a lower probability of consumption.

**Table 4:** Results of the consumption probability model for three selected food items with varying information included: mean absolute deviations (MAD), standard deviation of absolute deviations (SD) and squared correlation coefficients of probability estimate and true probability (Corr).

Model	Number of 24 h-FL	Tomatoes			Grapes			Buttermilk		
		MAD	SD	Corr	MAD	SD	Corr	MAD	SD	Corr
FFQ only^*^	0	0.1474		0.5776	0.1283		0.5476	0.1030		0.4489
Covariates	2	0.1413	0.0014	0.7423	0.1219	0.0010	0.7284	0.0797	0.0008	0.6962
	3	0.1178	0.0008	0.8128	0.1018	0.0008	0.8012	0.0674	0.0006	0.7752
	4	0.1029	0.0007	0.8532	0.0891	0.0007	0.8436	0.0594	0.0005	0.8218
	5	0.0926	0.0006	0.8791	0.0803	0.0007	0.8706	0.0538	0.0005	0.8523
	6	0.0846	0.0006	0.8975	0.0735	0.0005	0.8905	0.0495	0.0005	0.8742
	8	0.0734	0.0005	0.9214	0.0638	0.0004	0.9159	0.0432	0.0003	0.9031
	10	0.0656	0.0004	0.9365	0.0572	0.0003	0.9320	0.0388	0.0003	0.9213
	12	0.0598	0.0004	0.9468	0.0522	0.0003	0.9428	0.0356	0.0003	0.9338
Covariates + FFQ	2	0.1151	0.0009	0.8087	0.1003	0.0007	0.7931	0.0702	0.0005	0.7468
	3	0.1012	0.0007	0.8506	0.0883	0.0006	0.8382	0.0617	0.0005	0.8042
	4	0.0919	0.0005	0.8774	0.0802	0.0006	0.8675	0.0557	0.0004	0.8403
	5	0.0846	0.0005	0.8960	0.0739	0.0005	0.8875	0.0511	0.0004	0.8654
	6	0.0786	0.0005	0.9100	0.0687	0.0005	0.9028	0.0475	0.0004	0.8838
	8	0.0697	0.0004	0.9289	0.0608	0.0004	0.9234	0.0420	0.0003	0.9089
	10	0.0631	0.0003	0.9415	0.0551	0.0003	0.9369	0.0381	0.0003	0.9252
	12	0.0580	0.0004	0.9503	0.0508	0.0003	0.9464	0.0351	0.0003	0.9365

^*^Squared partial correlation coefficient, baseline covariates included: age at examination, sex, education, smoking status and body mass index.

With four 24 h-FLs available and including the covariates and the FFQ information in the probability model, the squared correlation increased by about 0.30 (tomatoes), 0.33 (grapes) and 0.39 (buttermilk), respectively compared to the squared partial correlation of the FFQ only model.

Overall, the observed values for the mean of the absolute deviation (MAD) were smallest for the item buttermilk, as it was the item with the smallest mean consumption probability. All MAD values decreased with an increasing number of 24 h-FL. Specifically, the covariate- and FFQ-adjusted model demonstrated that a higher number of 24 h-FL replications led to a lower standard deviation of absolute deviations, resulting in increased precision.

**Figure 3:**
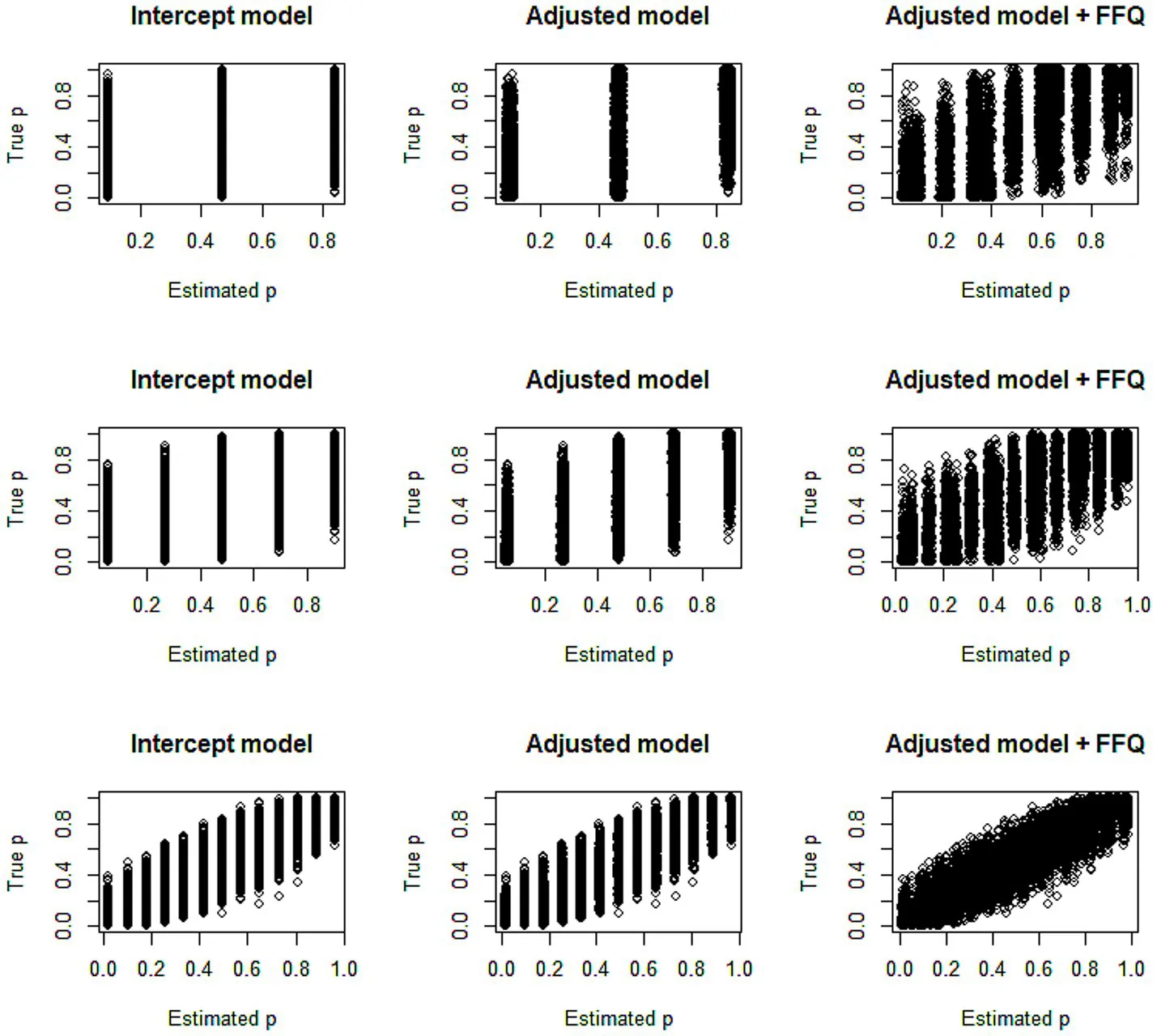
Example of effect of the number of 24-h food lists (24 h-FL), adjustment (age at examination, sex, education, smoking status, and body mass index) and the inclusion of FFQ information in estimating the probability of consumption with adapted MSM with 2, 4, and 12 24 h-FLs. First row shows estimates with two 24 h-FL available, middle row shows estimates with four 24 h-FL available, and last row shows estimates with 12 24 h-FL available.

Figure 3 illustrates the effect of the number of 24 h-FL and the inclusion of the FFQ when estimating the consumption probability for the first run of the simulation. The first column shows that, in the intercept (unadjusted) model, the estimated probabilities are limited to the number of 24 h-FL with consumption (0, 1, 2, etc.). While increasing the number of repetitions naturally narrowed the distribution along the diagonal of the true probability, the next two columns shows the effect of covariate adjustment as a small variation in the estimates close to the estimate of the intercept model, and additionally the stronger effect of the food frequency information, which leads to an even greater dispersion of the estimated values in direction of the true simulated probabilities.

## Discussion

The dietary intake data from the NAKO program provides the basis for research on the impact of habitual diet on health. This manuscript describes the method used to estimate usual dietary intakes for 152,304 participants. We adapted the MSM to estimate consumption probabilities and derive usual intakes for specific food items. We demonstrated that incorporating repeated 24 h-FLs improved consumption estimates and provided intake data for major food groups, energy, and macronutrients. These results were comparable to findings from the KORA study, supporting the validity of our approach. Furthermore, the sample with sufficient dietary information was comparable to the broader cohort, suggesting that missing data did not significantly introduce selection bias into the analyzed population.

A two-stage approach was used to model usual dietary intake in the large-scale NAKO dataset (438,166 24 h-FLs from 152,304 participants): First, consumption probabilities were estimated using repeated 24 h-FLs and FFQs; subsequently, consumption amounts were estimated using an external dataset. While LLMMs (27) are standard for repeated binary measurements of this type, their iterative optimization processes require significant computational resources, making them less practical for cohorts of this size. To ensure computational efficiency, we adapted the MSM (13, 28). Although previous simulations suggest that the original MSM may lead to biases when estimating episodically consumed foods (20), it provides unbiased estimates for frequently consumed foods (29, 30). Consequently, we modified the probability component of the MSM algorithm and could show that in the empirical comparison of the adapted MSM and LLMM showed a very high degree of agreement, suggesting the suitability of the MSM. Otherwise, the validity of the NVS II data was not validated, as it remains the only representative dataset available in Germany for this purpose.

While LLMMs are standard for repeated binary measurements, their iterative optimization process requires significant computational resources, making them less practical for cohorts of this size (involving over 150,000 clustered individuals and over 400,000 total 24 h-FLs). To ensure computational efficiency, we adapted the MSM method for the NAKO study because it bypasses this severe computational bottleneck while achieving high empirical agreement (squared correlations up to 0.99) with LLMM. However, it must be explicitly acknowledged that our empirical comparison validates the statistical agreement between the two statistical models, rather than the accuracy of usual dietary intake estimation.

The adapted MSM approach effectively produces measurement-error-corrected estimates that are suitable for diet-health outcome regressions via regression calibration (31). However, it is important to note, that because the shrinkage approach on residuals deliberately eliminates within-person variance these data should not be used to estimate population distribution tails, as the empirical tails are underrepresented (32).

Adjusting for socio-demographic and lifestyle covariates (e.g., BMI and smoking) can reduce confounding, account for systematic reporting biases, and explain sources of variability, thereby improving the precision of repeated-measurement models. However, these adjustments should be interpreted with care, as extensive covariate adjustment may attenuate genuine biological inter-individual variation, particularly when adiposity or specific lifestyle factors lie on the causal pathway between dietary exposures and metabolic disease outcomes. In such cases, adjustment may constitute over-adjustment, removing part of the biological effect of interest rather than simply reducing bias.

The estimated mean usual dietary intakes fall within reasonable ranges and show agreement with the German KORA FF4 study. Minor discrepancies likely reflect a more health-oriented NAKO subpopulation and broader temporal dietary shifts over the past two decades, such as documented increases in poultry and vegetable intake alongside a reduction in processed meat (33). Furthermore, NAKO’s slightly lower overall energy intakes, especially in women, stem from our deliberate identification and zero-imputation of true non-consumers using both the 24 h-FL and FFQ. Traditional models, such as the NCI method, lack this feature and artificially prevent zero-probability predictions.

Because participants provided varying numbers of 24 h-FLs (ranging from 1 to 4), macro- and food-group intake estimates were systematically lower when individuals with fewer completed records were integrated. To safeguard statistical power and prevent sample loss, we chose to include all eligible participants, but we advise researchers to conduct sensitivity analyses accounting for the number of 24 h-FL if quantitative intake precision is central to their study. In particular, projects using these generated data can carry out sensitivity analyses stratified by the number of completed 24 h-FLs (1 to 4), to ensure that the varying levels of detail in individual data do not, in themselves, influence observed associations between diet and health. Additionally, data credibility should be monitored by identifying potential misreporters through energy intake versus basal metabolic rate ratios derived from Schofield equations (34).

Our simulation study underscored that integrating FFQ frequency data improved the accuracy and correlation of individual consumption probabilities, yielding essential benefit when only one or two 24 h-FLs are available. This accuracy gain plateaued almost entirely at four 24 h-FLs. Similarly, Caroll et al. (4) showed that FFQs contribute important information in combination with repeated 24-h dietary recall data, especially in the case of episodically consumed foods or nutrients and recommended four to six administrations of 24-h dietary recalls should be considered along with administration of an FFQ in cohort studies.

Several limitations and key methodological assumptions of the study should be noted: Firstly, vegan and vegetarian meat and fish substitutes were underrepresented in the dietary assessment tools at the start of the study; however, this was corrected in the second follow-up examination.

Secondly, our methodology assumed that 24 h-FLs provide unbiased estimates of consumption probability; however, like all self-reported instruments, they remained subject to residual measurement error. Because true reference dietary assessment methods and recovery biomarkers for long-term consumption probabilities are unavailable, the repeated 24 h-FLs used in our proposed method currently serve as an applicable approximation.

Thirdly, pooling baseline and follow-up observations cross-sectionally assumed that the participants’ underlying consumption probabilities and dietary habits did not meaningfully change over the five-year gap. If true dietary shifts did occur during this period, treating these distinct time points as interchangeable repeated measures could artificially inflate within-person variance, introducing a measurement error that might attenuate subsequent diet-disease associations.

Fourthly, the estimation of daily intake levels was based on the assumption that external NVS II data (2005 and 2007) are directly applicable to the NAKO population (2014–2024). Given the long-term changes in dietary habits during this period, such as the increased consumption of vegetables and poultry and the decreased consumption of processed meat products, the application of these intake levels may lead to a systematic over- or underestimation of the absolute consumption of certain foods. Moreover, the NVS II remains currently the only comprehensive, nationally representative dietary survey for Germany.

Finally, the time period during which participants completed their questionnaires varied considerably due to the two survey waves; consequently, a specific variable is provided to record individual examination intervals so that researchers can account for this variation. Nevertheless, by opening the first follow-up examination to additional dietary data collection, NAKO succeeded in increasing the proportion of participants with at least three 24-h dietary records to 59%, which enabled a valid assessment of usual dietary intake for this large cohort. Overall, 25.6% of participants lacked a complete set of usable dietary data (at least one 24 h-FL and an FFQ), which could theoretically introduce selection bias if the missingness occurred non-randomly. However, because the demographic and lifestyle characteristics of the final analytical sample were highly comparable to those of the entire cohort, this missingness appeared to be at random and likely did not introduce substantial bias.

It should also be noted that the constraints of the MSM model and similar statistical models with regard to extremely rare (only episodically consumed) food items exhibiting high zero-inflation must be taken into account. The adapted MSM ensures general mathematical stability through the application of Firth’s logistic regression with an additional covariate (FLAC) (22) if convergence problems arise. Nevertheless, food items lying just above the “true zero” threshold may still be empirically under-represented at the extremes of the distribution.

## Conclusion

This study contributes to the planning and statistical analysis of large-scale studies involving dietary assessment by developing a computationally efficient methodology for estimating usual dietary intake using the adapted MSM method. A key finding for future study designs is the remarkable cost-effectiveness achieved through our combined data collection strategy: Incorporating a long-term FFQ into the statistical model reduces the need for an excessive number of short-term 24 h-FLs, thereby optimizing resource allocation and reducing the burden on participants. Our simulation study has shown that future follow-up waves of the NAKO, or the planning and conduct of other nutritional studies, could be expanded to include the same dietary assessment strategy comprising four (or more) 24 h-FLs combined with an FFQ supplemented by an external source for estimating daily consumption amounts. Having successfully generated measurement error-corrected estimates of dietary intake for over 150,000 participants, these estimates will serve as the basis for future investigations into the relationship between health and diet. The blended approach provides an applicable framework for investigating longitudinal diet-health relationships in nutritional epidemiology.

## Data Availability

The datasets presented in this article are not readily available because access to and use of NAKO data can be obtained via an electronic application portal (https://transfer.nako.de). Requests to access the datasets should be directed to NAKO e.V., Am Taubenfeld 21/2, 69123 Heidelberg, Germany; Link: https://transfer.nako.de.

## Footnotes

1
https://sms.dife.de/tool/sv24/en

2
https://sms.dife.de/tool/ffq2/en
