# Supplementary material for: Usual dietary intake estimation in the German National Cohort (NAKO)
Source: Front Nutr. 2026 Aug 26;13:1894787. doi: 10.3389/fnut.2026.1894787 (PMC13553502; doi:10.3389/fnut.2026.1894787)
Supplement: Supplementary file 1 [file Supplementary_file_1.docx]

**Usual dietary intake estimation in the German National Cohort (NAKO)**

Nina Wawro, Sylvia Gastell, Rafael Mikolajczyk, Nadine Glaser, Sabrina Schlesinger, Tamara Schikowski, André Karch, Henning Teismann, Nadia Obi, Volker Harth, Katharina S.Weber, Cara Övermöhle, Michael Leitzmann, Beate Fischer, Tobias Pischon, Katharina Nimptsch, Börge Schmidt,  Henry Völzke, Till Ittermann,  Annette Peters, Barbara Thorand,  Antje Hebestreit, Maike Wolters, Verena Katzke, Stefanie Jaskulski, Peggy Sekula, Lilian Krist, Stefan N. Willich, Bernd Holleczek, Michael Hoffmeister, Carolina Klett-Tammen, Dörthe Meyerdierks, Kerstin Wirkner, Johanna Conrad, Ute Nöthlings, Matthias B. Schulze, Jakob Linseisen, Sven Knüppel

[1.	Estimation of within- and between variance components	2](#_Toc235651137)

[2.	R code for adapted probability part of the Multiple Source Method (MSM)	3](#_Toc235651138)

[3.	SAS code for adapted probability part of the Multiple Source Method (MSM)	6](#_Toc235651139)

[4.	Table S1: Food items categorised according to the EPIC-Soft main food groups and food groups.	16](#_Toc235651140)

[5.	Code example for logistic linear mixed-effects model (LLMM) in R and SAS	19](#_Toc235651141)

[6.	Table S2: Usual dietary intake [g/day] by number of available 24-hour food lists (24h-FLs)	20](#_Toc235651142)

[7.	Figure S1: Applying MSM and LLMM on three selected food items for 5000 random selected participants. The left panel shows a scatter plot of estimated probabilities by MSM and LLMM (the grey line indicates the 45-degree line). The right panel shows the density curves (kernel type) of the estimated consumption probabilities.	21](#_Toc235651143)

# 1. Estimation of within- and between variance components

To estimate within- and between variance components we apply the following approach (*Rosner B. Multisample Inference. in Rosner B. Fundamentals of Biostatistics. Boston:Brooks/Cole, Cengage Learning, 2016, p. 605-606*):

Suppose we have the following random-effects one-way analysis-of-variance model: $r_{ij}=t_{i}+u_{ij}$, for$i=1, ..., n$, and $, j =1, ..., n_{i}$, where $r_{ij}$ = $j$th residual for the $i$th individual assuming that $t_{i}\sim N\left( 0, \sigma_{t}^{2} \right)$ is a random variable representing between-subject variability, and that $u_{ij}\sim N\left( 0, \sigma_{u}^{2} \right)$ is a random variable representing within-subject variability.

(1) We can compute the between mean square (MS) and within mean square (MS) as follows:

between mean square (MS) = $\frac{1}{k-1}\sum_{i=1}^{n} n_{i}\left( \overline{r}_{i}-{\overline{\overline{r}}}_{i} \right)$, where $\overline{r}_{i}=\frac{1}{n_{i}}\sum_{j=1}^{n_{i}} r_{ij}$ and ${\overline{\overline{r}}}_{i}=\frac{1}{M}\sum_{i=1}^{n} \sum_{j=1}^{n_{i}} r_{ij}$ and within mean square (MS) = $\frac{1}{N-k}\sum_{i=1}^{n} \sum_{j=1}^{n_{i}} \left( r_{ij}-\overline{r}_{i} \right)^{2}$, where M = $\sum_{i=1}^{n} n_{i}$.

(2) The within-variance component ($\sigma_{u}^{2}$) is estimated using the within MS

$\sigma_{u}^{2}$= within MS.

(3) The between-variance component ($\sigma_{t}^{2}$) is estimated by

$\sigma_{t}^{2}=max\left( 0, \frac{between MS - within MS}{n_{0}} \right)$, where $n_{o}=\frac{1}{n-1}\left( \sum_{i=1}^{n} n_{i}-\frac{\sum_{i=1}^{n} n_{i}^{2}}{\sum_{i=1}^{n} n_{i}} \right)$.

In a balanced design, the number of residuals for each individual are equal $(n_{1}=n_{2}=...=n_{n})$, resulting in $n_{0}=n$.

# 2. R code for adapted probability part of the Multiple Source Method (MSM)

```
library(tidyverse)
library(lme4)
library(reshape2)


# Function: msm_prob_r
# Author: Sven Knüppel
# Arguments
# data            data set, long-format, one row represents one observation
# outcome         string, represents the outcome variable (binary, 0 = no
#                 consumption, 1 = consumption), left side of the model equation
# pred_var        sting, formula of predictor variables, e.g. x1 + x2,
#                 right side of the model equation
# subj            string/numeric Identifier of subjects (repeated measurement)
# link            default: logit, logit or probit
#
# Value
# list of two elements
# 1. Individual estimated probabilities, tibble data set
# 2. msm_model  glm object of logistic regression

msm_prob_r <- function(data, outcome, pred_var, subj, link = "logit") {
  library(tidyverse)
  library(logistf)

  # Model formula
  formula <- paste0(outcome, " ~ ", pred_var)
  glmformula <- as.formula(formula)

  cols_needed <- unique(c(subj, all.vars(glmformula)))
  tmp_data <- tibble::as_tibble(na.omit(data[, cols_needed]))

  # Fit logistic regression model
  model <- glm(formula = glmformula,
               data = tmp_data,
               family = binomial(link = link))

  if (!(model$converged)) {
    cat("Convergence problems: converged = ", model$converged, "\n")
    cat("Firth's logistic regression with added covariate (FLAC) is used.\n")
    # Reference: Puhr R et al. (2017). Statistics in Medicine 36: 2302-2317.
    model <- logistf::flac(glmformula, data = tmp_data)
  }

  # Extract data for MSM output
  predicted <- predict(model, type = "response")

  tmp_data <- tmp_data %>%
    mutate(p = predicted, R = !!sym(outcome) - p)
  long_data <- tmp_data[, c(subj, "R")]

  ym <- long_data %>%
    group_by(!!sym(subj)) %>%
    summarise(y_m = mean(R),
              y_var = var(R),
              ni = n()) %>%
    ungroup()

  m_yi <- ym$y_m

  # Overall mean (grand mean)
  mm_y <- as.numeric(tmp_data %>%
                       summarise(mean_value = mean(R, na.rm = TRUE)))

  ni <- ym$ni
  k <- length(ni)
  n0 <- (sum(ni) - sum(ni ^ 2) / (sum(ni))) / (k - 1)
  n <- nrow(tmp_data)  # number of observations

  between_ss <- sum(ym$ni * (ym$y_m) ^ 2 - sum(tmp_data[, "R"]) / n)
  between_ms <- between_ss / (k - 1)

  within_ss <- sum(ym$y_var * (ym$ni - 1), na.rm = TRUE)
  within_ms <- within_ss / (n - k)

  F <- between_ms / within_ms

  s2 <- within_ms  # within-group variance

  # negative variance?
  if (between_ms < within_ms) {
    cat("Negative variance: use of method proposed by Hartung (1981)\n")
    # PSD-MINQMBE
    # positive semidefinite minimum norm quadratic minimum bias estimator
    # Hartung (1981).The Annals of Statistics;9(2):278-292.
    # Hartung & Voet (1986).JASA;81(395):689-691.
    s2_a <- n0 / (1 + (n0 ^ 2)) * between_ms
  } else {
    s2_a <- max(0, (between_ms - within_ms) / n0)  # between-group variance
  }

  kappa <- ifelse(s2_a / (s2_a + s2 / ni) <= 0, 0, (s2_a / (s2_a + s2 / ni)))

  # Shrinkage
  fR <- mm_y + kappa * (m_yi  - mm_y)

  result <- list(
    indiv_pred = as_tibble(
      tmp_data %>%
        group_by(!!sym(subj)) %>%
        summarise(pred = mean(p)) %>%
        ungroup() %>%
        mutate(
          fR = fR,
          msm_prob = pred + fR,
          kappa = kappa,
          grand_mean = mm_y
        ) %>% select(-pred, -fR)
    ),
    msm_model = model
  )

  return(result)
}

# simple simulation of repeated administration of 24-hour food list --------

set.seed(987)

n_subj <- 5000       # Number of participants
n_24hFL <- 4         # Number of 24-hour food lists
median_prob <- 0.05  # Median food item consumption probability

x1 <- rnorm(n_subj, mean = 0, sd = 2)
x2 <- rnorm(n_subj, mean = 0, sd = 2)
x3 <- rnorm(n_subj, mean = 0, sd = 2)

z <- log(median_prob / (1 - median_prob)) + log(1.15) * x1 - log(1.15) * x2
p <- 1 / (1 + exp(-z))

simulation <-
  tibble(
    subj = rep(1:n_subj, each = n_24hFL),
    sim_prob = rep(p, each = n_24hFL),
    day = rep(1:n_24hFL, n_subj),
    y = rbinom(
      n_subj * n_24hFL,
      prob = rep(p, each = n_24hFL),
      size = 1
    ),
    x1 = rep(x1, each = n_24hFL),
    x2 = rep(x2, each = n_24hFL),
    x3 = rep(x3, each = n_24hFL)
  )

# MSM
res_msm <-  msm_prob_r(simulation, "y", "x1 + x2 + x3", "subj", link = "logit")

res_msm_n <- res_msm$indiv_pred #%>%
  #rename(msm_prob = msm_prob)

# Logistic mixed-effects model
fglmer <- "y ~ x1 + x2 + x3 + (1 | subj)"
m_glmer <- lme4::glmer(
  fglmer
  ,
  data = simulation
  ,
  control = glmerControl(optimizer = "bobyqa")
  #, verbose = 1
  ,
  nAGQ = 64
  ,
  family = binomial(link = "logit")
)
res_glmer <- tibble(simulation[, "subj"], glmer_prob = predict(m_glmer, type = "response"))

# Merge results
res_glmer <- res_glmer %>%
  distinct(.keep_all = TRUE)

sim_prob <- simulation %>%
  select(subj, sim_prob) %>%
  distinct(.keep_all = TRUE)

res <- left_join(res_msm_n[, c("subj", "msm_prob")], res_glmer, by = "subj") %>%
  left_join(sim_prob, by = "subj")

res_long <- melt(res[, c("subj", "msm_prob", "glmer_prob", "sim_prob")], id = "subj")

# Density plots
res_long %>%
  ggplot(aes(x = value, color = variable)) +
  geom_density(lwd = 1.5, linetype = 1) +
  scale_color_grey() +
  theme_bw()

# Deviation estimates to simulated "true" probabilities
round(summary((res$sim_prob - res$msm_prob)), 5)
round(summary((res$sim_prob - res$glmer_prob)), 5)

# Correlation between estimates and simulated "true" probabilities
cor(res[, -1], method = "pearson")
cor(res[, -1], method = "spearman")
```

# 3. SAS code for adapted probability part of the Multiple Source Method (MSM)

```
/***********************************************************************************
 *
 * SAS Macro helper deleteDataSet
 * Author: Sven Knüppel
 *
 **********************************************************************************/;
%macro deleteDataSet(lib, name);
  %if &name = 'del_all_files' %then
    %do;

      proc datasets library = &lib kill;
      run;

    %end;
  %else
    %do;
      %if %sysfunc(exist(&lib..&name)) %then
        %do;

          proc datasets library = &lib. nolist memtype = data;
            delete &name.;
          run;

        %end;
      %else;/* %put Data set &lib..&name does not exist.;*/
    %end;
%mend deleteDataSet;

/***********************************************************************************
 *
 * SAS Macro helpers for MSM probability estimate
 * Author: Sven Knüppel
 *
 **********************************************************************************/;
%macro msm_prob_logistic(
      outcome =,
      pred_var =,
      class =,
      subj_id =,
      link =
      );
  title;
  title "MSM: Logistic regression (link = &link): &outcome.(event = 1) ~ &pred_var. (dataset: &data.)";
  ods output
    Association = msm_association
    FitStatistics = msm_fitstatistics
    RSquare = msm_partfitstats
    ConvergenceStatus = msm_cs
    ParameterEstimates = msm_params;

  proc logistic data = __data__;
    id &subj_id. &outcome. &pred_var.;
    class &class.;
    model &outcome.(event = "1") = &pred_var. /
      technique = newton
      lackfit
      rsquare
      link = &link.;
    output out = __logistic__ pred = glm_pred;
  run;

  data msm_cs;
    retain model;
    length model $20.;
    set msm_cs;
    model = "Logistic regression";
  run;

  /*"MSM: Tjur R2 for logistic regression (link = &link):
  &outcome.(event = 1) ~  &pred_var. (dataset: &data.)";
  * more information: https://statisticalhorizons.com/r2logistic:
  The definition is very simple. For each of the two categories of
  the dependent variable, calculate the mean of the predicted probabilities
  of an event. Then, take the difference between those two means. That s it!;
  proc ttest data = __logistic__;
  class &outcome.;
  var glm_pred;
  run;
  or use SQL
  */
  proc sql noprint;
    select mean(glm_pred) into: mean_pred
      from __logistic__;
  quit;

  proc sql noprint;
    select mean(glm_pred) into: mean_pred0
      from __logistic__
        where &outcome. = 0;
  quit;

  proc sql noprint;
    select mean(glm_pred) into: mean_pred1
      from __logistic__
        where &outcome. = 1;
  quit;

  data msm_tjur_R2_;
    retain label3 nValue3 label4 nValue4 label5 nValue5 label6 nValue6;
    label3 = "mean pred";
    nValue3 = &mean_pred.;
    label4 = "mean pred0";
    nValue4 = &mean_pred0.;
    label5 = "mean pred1";
    nValue5 = &mean_pred1.;
    label6 = "Tjur R2";
    nValue6 = nValue5 - nValue4;
  run;

  data msm_partfitstats;
    merge msm_partfitstats(drop = cvalue1 cvalue2)
      msm_tjur_R2_;
  run;

  %deleteDataSet(work, msm_tjur_R2_);
  title;
%mend msm_prob_logistic;

%macro msm_prob_flac(
      outcome =,
      pred_var =,
      class =,
      subj_id =,
      link = logit
      );
  title;
  title "MSM: Logistic regression FIRTH (link = &link): &outcome.(event = 1) ~  &pred_var. (dataset: &data.)";

  proc logistic data = __data__;
    id &subj_id. &outcome. &pred_var.;
    class &class.;
    model &outcome.(event = "1") = &pred_var. /
      firth
      technique = newton
      lackfit
      rsquare
      link = &link.;
    output out = __logistic2__(keep = &subj_id. &outcome. &pred_var. h_firth)
      h = h_firth;
  run;

  data __logistic2__;
    set __logistic2__;
    temp_pseudo = 0;
    temp_neww = 1;
    y = &outcome.;
    keep_id = 1;
    output;
    temp_pseudo = 1;
    temp_neww = h_firth / 2;
    y = &outcome.;
    keep_id = 0;
    output;
    temp_pseudo = 1;
    temp_neww = h_firth / 2;
    y = 1 - &outcome.;
    keep_id = 0;
    output;
  run;

  title "MSM: surveylogistic FLAC (link = &link): &outcome.(event = 1) ~  &pred_var. (dataset: &data.)";
  ods output
    Association = msm_association
    FitStatistics = msm_fitstatistics
    RSquare = msm_partfitstats
    ConvergenceStatus = msm_cs
    ParameterEstimates = msm_params;

  proc surveylogistic  data = __logistic2__;
    class &class.;
    model y(event = "1") = &pred_var. temp_pseudo /

      rsquare
      link = &link.
      technique = newton
      ridging = none;
    weight temp_neww;
    output out = __logistic__ (where = (keep_id = 1))
      pred = glm_pred;
  run;

  data msm_cs;
    retain model;
    length model $20.;
    set msm_cs;
    model = "FLAC";
  run;

  /*"MSM: Tjur R2 for logistic regression (link = &link):
  &outcome.(event = 1) ~  &pred_var. (dataset: &data.)";
  * more information: https://statisticalhorizons.com/r2logistic:
  The definition is very simple. For each of the two categories of
  the dependent variable, calculate the mean of the predicted probabilities
  of an event. Then, take the difference between those two means. That s it!;
  proc ttest data = __logistic__;
  class &outcome.;
  var glm_pred;
  run;
  or use SQL
  */
  proc sql noprint;
    select mean(glm_pred) into: mean_pred
      from __logistic__;
  quit;

  proc sql noprint;
    select mean(glm_pred) into: mean_pred0
      from __logistic__
        where &outcome. = 0;
  quit;

  proc sql noprint;
    select mean(glm_pred) into: mean_pred1
      from __logistic__
        where &outcome. = 1;
  quit;

  data msm_tjur_R2_;
    retain label3 nValue3 label4 nValue4 label5 nValue5 label6 nValue6;
    label3 = "mean pred";
    nValue3 = &mean_pred.;
    label4 = "mean pred0";
    nValue4 = &mean_pred0.;
    label5 = "mean pred1";
    nValue5 = &mean_pred1.;
    label6 = "Tjur R2";
    nValue6 = nValue5 - nValue4;
  run;

  data msm_partfitstats;
    merge msm_partfitstats(drop = cvalue1 cvalue2)
      msm_tjur_R2_;
  run;

  %deleteDataSet(work, msm_tjur_R2_);
  %deleteDataSet(work, __LOGISTIC2__);
  title;
%mend msm_prob_flac;

**********************************************************************************
* SAS Macro: msm_prob
* Auhtor: Sven Knüppel
**********************************************************************************;

* Input parameter
* ----------------
* data     SAS dataset        long-format, one row represents one observation
* outcome  character          represents the outcome variable (binary, 0 = no
*                             consumption, 1 = consumption), left side of the
*                             model equation
* pred_var  character (list)  list of predictor variables, right side of the
*                             model equation
* subj_id   character/numeric Identifier of subjects (repeated measurement)
* link      character         default: logit, logit or probit
**********************************************************************************;

* Output datasets (chronological order)
* -------------------------------------
* msm_association   Association of predicted probabilities and observed responses
* msm_cs            Convergance status after running proc logistic
* msm_fitstatistics Likehood statistics
* msm_params        Maximum likelihood estimates of model parameters
* msm_partfitstats  pseudo-R2 vlaues, Tjur R2
* msm_prob          Individual estimated probabilities
**********************************************************************************;
%macro msm_prob(
  data =,
  outcome =,
  pred_var =,
  class =,
  subj_id =,
  link =   /*logit oder probit wird beim Aufruf übergeben*/
);
  options nonotes;
  title;
  %local _timer_msm;

  /* Start timer */
  %let _timer_msm = %sysfunc(datetime());

  data _null_;
    timer_msm = &_timer_msm;
    put 60*'-' /
      " MACRO MSM STARTS:  " timer_msm datetime. /
      " USER:              &SYSUSERID." /
      " SAS VERSION:       &SYSVER." /
      " SAS VERSION(long): &SYSVLONG4." /
      " OPERATING SYSTEM:  &SYSSCP. (&SYSSCPL.)" / 60*'-';
  run;

  title;

  *start_timer;
  data __data__;
    retain &subj_id. &outcome. &pred_var. &class.;
    set &data.;
    FORMAT _all_;
    keep &subj_id. &outcome. &pred_var. &class.;
  run;

  proc datasets library = work nolist;
    modify __data__;
    attrib _all_ label = '';
  quit;

  %msm_prob_logistic(
    outcome = &outcome.,
    pred_var = &pred_var.,
    class = &class.,
    subj_id = &subj_id.,
    link = &link.
    );

  /* check statistical poperties;*/
  proc sql noprint;
    select status into: status
      from MSM_CS;
  quit;

  %if &status. ne 0 %then
    %do;
      /* write to log file */
      data _null_;
        put " Convergence problems: status = %trim(&status.).";
        put " Firth's logistic regression with added covariate (FLAC) is used.";
      run;

      /*Write to the output */
      data _null_;
        file print;
        put " Convergence problems: status = %trim(&status.).";
        put " Firth's logistic regression with added covariate (FLAC) is used.";
      run;

      %msm_prob_flac(
        outcome = &outcome.,
        pred_var = &pred_var.,
        class = &class.,
        subj_id = &subj_id.,
        link = &link.);
    %end;

  /* Mean values;*/
  proc sql noprint;
    select min(glm_pred) into: min_pred
      from __logistic__;
  quit;

  proc sql noprint;
    select max(glm_pred) into: max_pred
      from __logistic__;
  quit;

  run;

  proc sort data = __logistic__;
    by &outcome.;
  run;

  proc sgplot data = __logistic__ noautolegend;
    histogram glm_pred /  group = &outcome. name = "a" transparency = 0.5;
    density glm_pred / group = &outcome. type = kernel
      lineattrs = (pattern = solid);
    keylegend "a" / location = inside position = topright across = 1 noborder;
  run;

  proc sort data = __logistic__;
    by &subj_id.;
  run;

  /*title "MSM: Tjur R2 for logistic regression (link = &link): &outcome.(event = 1) ~  &pred_var. (dataset: &data.)";
   * more more information: https://statisticalhorizons.com/r2logistic:
  The definition is very simple. For each of the two categories of
  the dependent variable, calculate the mean of the predicted probabilities
  of an event. Then, take the difference between those two means. That s it!;
  proc ttest data = __logistic__;
  class &outcome.;
  var glm_pred;
  run;
  => the mean difference can be found on the output of hplogistic (using statement partition)
  */
  data __logistic__;
    set __logistic__;
    r = &outcome. - glm_pred;
    f_r = r;
  run;

  proc sql;
    create table __grand_mean__ as
      select mean(f_r) AS __grand_mean__
        from __logistic__;
  quit;

  data __grand_mean__;
    set __grand_mean__;
    call symput("__grand_mean__", __grand_mean__);
  run;

  data __logistic__;
    set __logistic__;
    f_r = f_r;
  run;

  proc sort data = __logistic__;
    by &subj_id.;
  run;

  proc summary data = __logistic__;
    by &subj_id.;
    id &outcome.;
    output out = __phab__
      mean(&outcome.) = c
      mean(f_r) = frm
      var(f_r) = frV
      mean(glm_pred) = glm_pred
      n(f_r) = days;
  run;

  proc sql;
    create table __bcrm__ as
      select mean(frm) AS __bcrm__
        from __phab__;
  quit;

  data _null_;
    set __bcrm__;
    call symput("brcm", __bcrm__);
  run;

  proc sql;
    create table __nn__ as
      select count(*) AS __nn__
        from __phab__;
  quit;

  data _null_;
    set __nn__;
    call symput("__nn__", __nn__);
  run;

  proc sql;
    create table __total_obs__ as
      select count(*) AS __total_obs__
        from __logistic__;
  quit;

  data _null_;
    set __total_obs__;
    call symput("__total_obs__", __total_obs__);
  run;

  data __phab__;
    set __phab__;
    __bcrm__ = &brcm.;
    __nn__ = &__nn__.;
    days2 = days * days;
    __b_ms__ = days * (frm - __bcrm__) **2 / (__nn__ - 1);
  run;

  proc sql;
    create table __b_ms__ as
      select sum(__b_ms__) AS __b_ms__
        from __phab__;
  quit;

  data _null_;
    set __b_ms__;
    call symput("between_ms", __b_ms__);
  run;

  proc sort data = __logistic__;
    by &subj_id.;
  run;

  proc sort data = __phab__;
    by &subj_id.;
  run;

  data __CMEANS__;
    merge __logistic__(keep = &subj_id. f_r) __phab__(keep = &subj_id. frm __nn__);
    __total_obs__ = &__total_obs__.;
    __w_ms__ = (f_r - frm)**2 / (__total_obs__ - __nn__);
    by &subj_id.;
  run;

  proc sql;
    create table __w_ms__ as
      select sum(__w_ms__) AS __w_ms__
        from __CMEANS__;
  quit;

  data _null_;
    set __w_ms__;
    call symput("within_ms", __w_ms__);
  run;

  proc sql;
    create table __ndays__ as
      select sum(days) AS __ndays__
        from __phab__;
  quit;

  data _null_;
    set __ndays__;
    call symput("__ndays__", __ndays__);
  run;

  proc sql;
    create table __ndays2__ as
      select sum(days2) AS __ndays2__
        from __phab__;
  quit;

  data _null_;
    set __ndays2__;
    __ndays__ = &__ndays__.;
    __nn__ = &__nn__.;
    n0 = (__ndays__ - __ndays2__ / __ndays__) / (__nn__ - 1);
    between_ms = &between_ms.;
    within_ms = &within_ms.;

    /* assuming positive variance component*/
    msm_p_s2u = (between_ms - within_ms) / n0;

    /* negative variance? */
    if (between_ms < within_ms) then
      do;
        /**** PSD-MINQMBE ****/
        /*    positive semidefinite minimum norm quadratic minimum bias estimator */
        /*    Hartung (1981) The Annals of Statistics;9(2):278-292. */
        /*    Hartung & Voet (1986) JASA;81(395):689-691. */
        msm_p_s2u = n0 / (1 + (n0 **2)) * between_ms;
      end;

    call symput ("msm_p_s2u", msm_p_s2u);
    call symput ("msm_p_s2e", within_ms);
  run;

  %let neg_var = %sysevalf(&between_ms. - &within_ms.);

  %if %sysevalf(&neg_var. < 0, boolean) %then
    %do;
      %put "Negative variance: use of method proposed by Hartung (1981)";
    %end;

  data msm_cs;
    set msm_cs;
    negative_variance = %if %sysevalf(&neg_var. < 0, boolean) %then

      %do;
        1
      %end;
  %else
    %do;
      0
    %end;;

  residuals_grand_mean = &__grand_mean__.;
  run;

  data msm_prob;
    set __phab__;
    __grand_mean__ =  &__grand_mean__.;
    msm_p_s2u = &msm_p_s2u.;
    msm_p_s2e = &msm_p_s2e.;
    kappa = (msm_p_s2u / (msm_p_s2u + msm_p_s2e / days));
    r_shrink = __grand_mean__  + kappa * (frm - __grand_mean__);
  run;

  data msm_prob;
    retain &subj_id. days c &outcome. msm_prob;
    set msm_prob;
    err_prob = 0;
    drop __b_ms__ __bcrm__ __grand_mean__ __nn__  _type_ _freq_ days2 frm frV r_shrink;

    * err_prob is 1 in case of msm_prob > 0 and -1 in case of msm_prob < 0;
    msm_prob = glm_pred + r_shrink;

    if (msm_prob > 1) then
      do;
        msm_prob = 1;
        err_prob = 1;
      end;

    if (msm_prob < 0) then
      do;
        msm_prob = 0;
        err_prob = -1;
      end;
  run;

  proc sql noprint;
    select min(msm_prob) into: min_msm
      from msm_prob;
  quit;

  proc sql noprint;
    select max(msm_prob) into: max_msm
      from msm_prob;
  quit;

  run;

  data _null_;
    file print notitles;
    put 60*'-';
    put "Minimum of predicted probabilities (log reg): &min_pred.";
    put "Maximum of predicted probabilities (log reg): &max_pred.";
    put;
    put "Minimum of MSM predicted probabilities (MSM): &min_msm.";
    put "Maximum of MSM predicted probabilities (MSM): &max_msm.";
    put 60*'-';
  run;

  proc sort data = msm_prob;
    by &outcome.;
  run;

  proc sgplot data = msm_prob noautolegend;
    histogram msm_prob;
    density msm_prob / type = kernel
      lineattrs = (pattern = solid);
  run;

  proc sort data = msm_prob;
    by &subj_id.;
  run;

  %deleteDataSet(work, __data__);
  %deleteDataSet(work, __logistic__);
  %deleteDataSet(work, __PHAB__);
  %deleteDataSet(work, __b_ms__);
  %deleteDataSet(work, __w_ms__);
  %deleteDataSet(work, __bcrm__);
  %deleteDataSet(work, __CMEANS__);
  %deleteDataSet(work, __grand_mean__);
  %deleteDataSet(work, __ndays__);
  %deleteDataSet(work, __ndays2__);
  %deleteDataSet(work, __nn__);
  %deleteDataSet(work, __total_obs__);

  /* Stop timer */
  data _null_;
    timer_stop = datetime();
    dur = timer_stop - &_timer_msm;
    put 60*'-' /
      ' MACRO MSM STOPS:    ' timer_stop datetime. /
      ' TOTAL DURATION: ' dur time14.2 ' (hh:mm:ss.ss) ' / 60*'-';
  run;

  title;
  options notes;
%mend msm_prob;

/* Simple simulation to apply MSM method comared to logistic linear mixed effects model (LLMM) */

/*
 *If needed, clear work;
proc datasets library = work kill;
run;
quit;
*/
data simulation;
  call streaminit(987);
  median_prob = 0.05;

  * Median food item consumption probability;
  n_24hFL = 4;

  * Number of 24-hour food lists (24h-FL);
  do subj = 1 to 5000;
    * Number of participants;
    x1 = rand("normal", 0, 2);

    * Covariates;
    x2 = rand("normal", 0, 2);
    x3 = rand("normal", 0, 2);
    z =  log(median_prob / (1 - median_prob)) + log(1.15) * x1 - log(1.15) * x2;
    sim_prob = 1 / (1 + exp(-z));

    * simulated 'true' frequency;
    do day = 1 to n_24hFL;
      y = rand("binomial", sim_prob, 1);

      * 1 = consumption or 0 = no consumption on day;
      output;
    end;
  end;

  drop median_prob n_24hFL;
run;

proc means data = simulation mean median;
  var sim_prob y;
run;

* Multiple Source method - probability part;
%msm_prob(
  data = simulation,
  outcome = y,
  pred_var = x1 x2 x3,
  class =,
  subj_id = subj,
  link = logit
  );

* Logistic linear mixed effects model(LLMM);
proc glimmix data = simulation method = quad(qmax = 64) noclprint;
  class subj;
  model y = x1 x2 x3 / s dist = binomial link = logit;
  random intercept / subject = subj;
  output out  = glmix_prob pred(ilink) = glmix_prob;
run;

proc sort data = simulation out = simp nodupkey;
  by subj sim_prob;
run;

proc sort data = msm_prob;
  by subj;
run;

proc sort data = glmix_prob nodupkey;
  by subj glmix_prob;
run;

data res;
  merge
    simp(keep = subj sim_prob)
    msm_prob(keep = subj msm_prob)
    glmix_prob(keep = subj glmix_prob);
  label glmix_prob = " ";
  diff_sim_msm = (msm_prob - sim_prob);
  diff_sim_glmix = (msm_prob - glmix_prob);
  by subj;
run;

proc means data = res n nmiss mean std min p25 p50 p75 max;
  var sim_prob msm_prob glmix_prob diff_sim_msm diff_sim_glmix;
run;

proc corr data = res pearson spearman;
  var sim_prob msm_prob glmix_prob;
run;

proc sgplot data = res noautolegend;
  series x = msm_prob y = msm_prob / lineattrs = (color = red thickness=2);
  series x = glmix_prob y = glmix_prob / lineattrs=(color = red thickness = 2);
  scatter x = msm_prob y = glmix_prob;
run;

proc sgplot data = res;
  density sim_prob / type = kernel legendlabel = "SIM";
  density msm_prob / type = kernel legendlabel = "MSM";
  density glmix_prob / type = kernel legendlabel = "LLMM";
run;
```

# 4. Table S1: Food items categorised according to the EPIC-Soft main food groups and food groups.

| **EPIC-Soft main food groups** | **EPIC-Soft food groups** | **Food Items** |
| --- | --- | --- |
| **01 Potatoes and other tubers** | 0101 Potatoes | Potatoes, Others, Mashed potatoes, Potato dumplings, Home fries, Chips, Potato pancake, Potato salad, Filled potato pockets |
| **02 Vegetables** | 0200 Vegetable | Asparagus, Other vegetables, Other raw vegetables |
|  | 0201 Leafy greens (except cabbage) | Light and dark green lettuce, Spinach |
|  | 0202 Fruit vegetables | Tomatoes, Cucumber, Pepper, Gherkin, Avocado, bell pepper, Zucchini, Aubergine |
|  | 0203 Root vegetable | Carrots raw |
|  | 0204 Cabbage vegetables | Stem cabbage, Sauerkraut, Cooked white cabbage, Green cabbage, Broccoli, Cauliflower, Brussels sprouts, Red cabbage |
|  | 0205 Mushrooms | Mushrooms |
|  | 0207 Onions, garlic | Garlic, raw, cooked, roasted, pickled, Onions |
|  | 0209 Mixed salads, mixed vegetables | Vegetable mixes |
| **03 Legumes** | 0301 Legumes | Legumes (e.g peas, beans, chickpeas) |
| **04 Fruits, nuts and seeds** | 0400 Fruits | Dried fruits |
|  | 0401 Fruits | Apple, Others, Pear, Peach, Apricot, Nectarine, Orange, Tangerine, Raspberries, Kiwi, Gooseberries, Fresh fig, Pineapple, Mirabelle, Cape gooseberry, Pomegranate, Cherries, Plum, Grapes, Strawberries, Blueberries, Melon, Banana, Citron |
|  | 0402 Nuts, seeds, nut spread | Nuts (salted, fresh, e.g. hazelnuts, walnuts, almonds, pine nuts, pistachio), Others, Seeds (e.g. flaxseed, sesame), Seeds (e.g. sunflower seed, pumpkin seed), Trail mix |
|  | 0403 Mixed Fruits | Cold sweet soup with fruits, Rødgrød, Stewed fruit, Fruit salad, Fresh fruit salad, Stewed fruit and canned fruit, Fruit jelly, Red fruit pudding |
|  | 0404 Olives | Olives |
| **05 Dairy products** | 0500 Dairy products | Sour milk cheese, Other milk products, Other quark and cheese |
|  | 0501 Milk | Buttermilk, added milk (coffee), added milk (tea), added milk (decaffeinated coffee), Milk (1, 5% fat), Milk (0, 3% fat), 1% fat), Milk (fat unknown), Milk (3, 5%) |
|  | 0502 Milk beverages | Cocoa, Mixed milk beverages or milk shakes |
|  | 0503 Yoghurt | Fruit yoghurt (1.5% fat), Fruit yoghurt (10% fat), Fruit yoghurt (3.5% fat), Fruit yoghurt (0.3% fat), Fruit yoghurt (0.1% fat), Fruit yoghurt (fat unknown), Plain yoghurt (10% fat), Plain yoghurt (3,5% fat), Plain yoghurt (1.5% fat), Plain yoghurt (0,3% fat), Fruit quark, Plain yoghurt (0.1% fat), Sour and curdled milk, Kefir, Plain yoghurt (fat unknown) |
|  | 0504 Fromage blanc, petitis suisses | Curd with herbs, Herb quark, double cream level, Harb quark usual fat, Quark usual fat, Quark low fat, Quark and double cream level, Herb quark low fat, Herb quark (fat unknown), Quark (fat unknown) |
|  | 0505 Cheese (including fresh cheese) | Mozzarella, Feta, Soft cheese and havey cream level, Semi-hard cheese (fat unknown), Soft cheese low fat, Soft cheese (fat unknown), Semi-hard cheese low fat, Cream cheese (fat unknown), Cream cheese usual fat, Cream cheese low fat, Soft cheese usual fat, Cream cheese and double cream level, Semi-hard cheese usual fat |
|  | 0506 Cream desserts, pudding (milk-based) | Pudding |
|  | 050700 Dairy, non-diary creams | Whipped cream, Cream, Crème fraîche |
|  | 0508 Milk for coffee, creamer | with condensed milk, added condensed milk (coffee), added condensed milk (tea), added condensed milk (decaffeinated coffee) |
| **06 Cereals and cereal products** | 0600 Cereals, cereal products | Others, Grain burger, Veggie burger, Bruised grain |
|  | 0601 Flour, flakes, starches, semolina used as flour | Cereal flakes, Grain meal |
|  | 0602 Pasta, rice, other grain | Rice, Pasta, noodles |
|  | 060300 Bread, crispbread, rusks | Others, Dinkel wheat bread and buns, Lye pastries, Flatbread, Multigrain bread and buns, Rye bread, Toast bread, Brown bread, Wheat bread, Rye buns, Baguette |
|  | 060301 Bread | Flatbread, Dinkel wheat bread and buns, Lye pastries, Multigrain bread and buns, Wheat bread, Buns, Baguette, Rye bread, rye buns, Brown bread, Toast bread, Others |
|  | 0604 Breakfast cereals | Others, Corn flakes, Rice crispies |
|  | 0605 Salty biscuits, asperitif biscuits, crackers | Salty biscuits, Snacks |
|  | 0607 Whole grains (pasta, cereal, bread and toast) | Muesli, Whole grain bread and buns, Whole grain toast bread, Whole-grain noodles |
| **07 Meat and meat products** | 0700 Fresh meat | Others, Veal, Lamb, Venison, Other meat and meat products |
|  | 070100 Meat, meat products | Mixed ground meat, Bolognese sauce, Shashlik, Meat skewer, Gyros, Hamburger, Meat loaf, Pork, Roast pork, Beef roulade, Pork goulash, Beef, Beef goulash |
|  | 070101 Beef | Beef Roulade, Beef goulash, Beef, Gyros, Pork, Meat loaf, Shashlik, Roast pork, Bolognese sauce, Pork goulash, Hamburger, Meat skewer, Mixed ground meat |
|  | 070103 Pork | Roast pork, Pork goulash, Pork, Gyros, Meat loaf, Shashlik, Beef roulade, Bolognese sauce, Beef, Hamburger, Meat skewer, Beef goulash, Mixed ground meat |
|  | 070200 Poultry | Poultry meat cut into strips, Poultry |
|  | 0704 Processed meat | Meat salad, Liverwurst, Collared pork, Others, Salami, Chorizo, Bologna, Mortadella, Blood sausage, Boiled ham, Bacon (cured), Cabanossi, Poultry sausage, Aspic, Bavarian meat loaf, Bavarian veal sausage, Bratwurst, Boiled sausages, Wiener |
|  | 0705 Offals | Other offal, Liver |
| **08 Fish and shellfish** | 0800 Fish, Shellfish | Hering salad, Salmon, Others, Trout, Tuna, Coalfish, Codfish, "Schlemmerfilet", Fish sticks, Calamari, Shellfish, Mackerel, Herring, Soused herring (matjes), Fried herring, Pickled herring, Buckling (hot smoked herring), Sprat, Bristling, Eel |
| **09 Eggs and egg products** | 0900 Eggs, egg products | Egg salad, Others |
|  | 0901 Eggs | Boiled egg, Fried egg, Omelette |
| **10 Fats** | 1000 Fat | Ingredients for meat (Other fat, Bacon, Lard, I don’t know), Ingredients for fish (Other fat, Bacon, Lard, I don’t know), Ingredients for cooked vegetables (Other fat, Bacon, Lard, I don’t know), Ingredients for raw vegetables (Other, with raw vegetables) |
|  | 1001 Vegetable oils | Linseed oil, Canola oil, Safflower oil, Germ oil, Other vegetable oil, Grape seed oil, Olive oil |
|  | 1002 Butter | Ingredients for cooked vegetables (Butter), Ingredients for fish (Butter), Ingredients for meat (Butter), Spread on bread (Butter, Lard) |
|  | 1003 Margarines | Ingredients for fish (Margarine), Spread on bread (Margarine, low-fat margarine), Ingredients for cooked vegetables (Margarine), Ingredients for meat (Margarine) |
| **11 Sugar and confectionary** | 1100 Sugar, confectory | Others |
|  | 1101 Sugar, honey, jam | Marmalade, Jam, Honey, Sugar beet molasses, added honey (hot drinks), added sugar (hot drinks) |
|  | 1102 Chocolate, candy bars, confectionery | Nutella, Nougat cream, Chocolate, Chocolate candy, other chocolate or sweets with chocolate |
|  | 1103 Confectionery non chocolate | Other sweets without chocolate |
|  | 110500 Ice cream, water ice | Ice cream |
| **12 Cakes** | 1200 Cakes | Others, Waffles, Pancakes, Cake |
|  | 1201 Cake, Pâté | Croissant, Fruit cake, Tart, Pie, Yeast cake and pastry, Sponge cake, Cake with cream, Cake without filling, Muffins, Cheese cake, Tiramisu, Mousse au chocolate |
|  | 1202 Dry cakes, biscuits | Cookies or biscuits with chocolate icing, Cookies or biscuits without chocolate icing |
| **13 Non-alcoholic beverages** | 1300 Non-alcoholic beverages | Others, Non-alcoholic beer, Malt beer |
|  | 1301 Fruit and vegetable juices | Other vegetable juice, Apple juice, Orange juice, Grape juice, Grapefruit juice, Elder juice, Multivitamin juice, Other fruit juices, Tomato juice |
|  | 1302 Soft drinks | Lemonade, Cola, Diet Lemonade, Diet Coke |
|  | 130301 Coffee | Coffee, Espresso, Cappuccino, Caffè latte, Decaffinated coffee, Others |
|  | 130302 Tea | Black tea, Green tea, Others, Decaffinated coffee |
|  | 130303 Herbal tea | Others, Fruit tea, Herbal tea, Decaffinated coffee |
|  | 1304 Water | Mineral water, Tap water |
| **14 Alcoholic beverages** | 1401 Wine | White wine, Red wine, Rosé wine, Sparkling wine, Prosecco |
|  | 1403 Beer, cider | Beer, Weissbier, Light beer, low-alcohol beer, Bock, Malt liquor |
|  | 1404 Spirits, Brandy, Liqueurs | Liqueur, Spirits (Brandy, Whiskey, Vodka, Eau de vie, Rum, Korn) |
|  | 1407 Cocktails, punches | Others, Shandy (beer and lemonade), Radler, Mulled wine, Wine spritzer, Cocktails and other mixed alcoholic beverages |
| **15 Condiments and sauces** | 150100 Sauces | Others, Soy sauce, Other sauce with meat or fish, Other sauce with cooked vegetables, Other sauce with side dishes (with potatoes, pasta, rice), Ketchup |
|  | 150101 Tomato sauces | Tomato sauce, Ketchup |
|  | 150102 Dressing sauces | Yoghurt dressing, Vinegar and oil dressing, Vinegar oil herbal dressing, French dressing, Cocktail dressing, Ketchup |
|  | 150103 Mayonnaises and similars | Mayonnaise, Remoulade, Ketchup |
|  | 1504 Condiments | Mustard, Vinegar |
| **16 Soups and bouillon** | 1601 Soups | Others, Clear soup (e.g. vegetable soup or soup with noodles), Cream of vegetable soup (e.g. of broccoli or asparagus) |
| **17 Miscellaneous** | 1700 Miscellaneous | Others, Vegetarian bread spread |
|  | 1701 Soy products | Tofu (meat substitute), Soy milk |

# 5. Code example for logistic linear mixed-effects model (LLMM) in R and SAS

**R:**

library(lme4)

m_glmer <- lme4::glmer(

y ~ x1 + x2 + x3 + (1 | subj),

data = data,

control = glmerControl(optimizer = "bobyqa"),

nAGQ = 64,

family = binomial(link = "logit")

)

**SAS:**

proc glimmix

data = data method = quad(qmax = 64) noclprint;

class subj;

model y = x1 x2 x3 / s dist = binomial link = logit;

random intercept / subject = subj;

output out = glmix_prob pred(ilink) = pred_prob;

run;

# 6. Table S2: Usual dietary intake [g/day] by number of available 24-hour food lists (24h-FLs)

|  | **at least one**  **24h-FL + one FFQ** | | **at least two**  **24h-FL + one FFQ** | | **at least three**  **24h-FL + one FFQ** | | **at least four**  **24h-FL + one FFQ** | |
| --- | --- | --- | --- | --- | --- | --- | --- | --- |
| **Sample size** | **152.304** | | **124.799** | | **89.494** | | **71.569** | |
| **Main food group** | **mean** | **sd** | **mean** | **sd** | **mean** | **sd** | **mean** | **sd** |
| potatoes, other tuber | 51.8 | 18.3 | 51.9 | 18.3 | 52.1 | 18.3 | 52.3 | 18.4 |
| vegetables | 157.5 | 53.3 | 159.1 | 53.5 | 161.1 | 53.7 | 161.8 | 54.0 |
| legumes | 6.9 | 3.5 | 6.9 | 3.5 | 6.9 | 3.5 | 7.0 | 3.5 |
| fruits, nuts | 257.7 | 109.5 | 261.2 | 110.3 | 266.3 | 111.5 | 269.3 | 112.1 |
| milk products | 194.7 | 94.0 | 196.5 | 94.0 | 199.0 | 94.0 | 199.5 | 94.4 |
| cereals | 146.1 | 48.1 | 146.9 | 48.3 | 148.0 | 48.5 | 147.9 | 48.6 |
| meat and meat products | 77.7 | 43.6 | 77.1 | 43.1 | 76.4 | 42.7 | 75.8 | 42.4 |
| fish and shellfish | 18.5 | 12.3 | 18.5 | 12.4 | 18.7 | 12.5 | 18.8 | 12.6 |
| egg and egg products | 20.1 | 11.6 | 19.9 | 11.4 | 19.7 | 11.3 | 19.6 | 11.2 |
| fats | 15.5 | 6.6 | 15.6 | 6.6 | 15.9 | 6.6 | 16.0 | 6.7 |
| sugar and sweets | 33.0 | 16.1 | 33.4 | 16.2 | 34.0 | 16.5 | 34.2 | 16.6 |
| cake | 39.5 | 17.0 | 40.1 | 17.1 | 40.8 | 17.1 | 41.0 | 17.1 |
| non-alcoholic drinks | 1,970.5 | 364.4 | 1,982.7 | 363.0 | 1,997.5 | 360.8 | 2,000.2 | 360.9 |
| alcoholic drinks | 124.6 | 139.6 | 126.1 | 139.7 | 128.0 | 139.5 | 128.9 | 139.9 |
| condiments, sauces | 17.6 | 7.7 | 17.6 | 7.7 | 17.7 | 7.7 | 17.6 | 7.6 |
| soup, bouillon | 27.1 | 16.9 | 27.5 | 16.9 | 27.9 | 16.9 | 28.0 | 16.8 |
| other foods | 8.0 | 12.6 | 8.4 | 12.7 | 8.8 | 12.9 | 8.9 | 12.9 |
| total energy [kcal/day] | 1,762.50 | 432.1 | 1,775.50 | 432.3 | 1,793.00 | 432 | 1,796.80 | 433.2 |
| alcohol | 7.2 | 7.2 | 7.4 | 7.3 | 7.5 | 7.3 | 7.6 | 7.3 |
| fibre | 21.3 | 6.0 | 21.5 | 6.1 | 21.9 | 6.1 | 22.0 | 6.1 |
| protein | 62.7 | 16.0 | 63.0 | 15.9 | 63.4 | 15.9 | 63.4 | 15.9 |
| fat | 76.1 | 19.9 | 76.5 | 19.9 | 77.1 | 19.9 | 77.1 | 19.9 |
| carbohydrates | 189.2 | 47.7 | 191.0 | 47.8 | 193.4 | 48.0 | 194.0 | 48.1 |
| sd, standard deviation | | | | | | | | |

# 7. Figure S1: Applying MSM and LLMM on three selected food items for 5000 random selected participants. The left panel shows a scatter plot of estimated probabilities by MSM and LLMM (the grey line indicates the 45-degree line). The right panel shows the density curves (kernel type) of the estimated consumption probabilities.
